# A review on spectral data preprocessing techniques for machine learning and quantitative analysis

**DOI:** 10.1016/j.isci.2025.112759

**Published:** 2025-05-29

**Authors:** Chunsheng Yan

**Affiliations:** 1 Zhejiang University Library, Hangzhou 310058, China; 2 State Key Laboratory of Extreme Photonics and Instrumentation, Hangzhou 310058, China

**Keywords:** Physics, Computer science, Engineering

## Abstract

Spectroscopic techniques are indispensable for material characterization, yet their weak signals remain highly prone to interference from environmental noise, instrumental artifacts, sample impurities, scattering effects, and radiation-based distortions (e.g., fluorescence and cosmic rays). These perturbations not only significantly degrade measurement accuracy but also impair machine learning–based spectral analysis by introducing artifacts and biasing feature extraction. This review provides a systematic evaluation of critical spectral preprocessing methods—encompassing cosmic ray removal, baseline correction, scattering correction, normalization, filtering and smoothing, spectral derivatives, and advanced techniques like 3D correlation analysis—highlighting their theoretical underpinnings, performance trade-offs, and optimal application scenarios. The field is undergoing a transformative shift driven by three key innovations: context-aware adaptive processing, physics-constrained data fusion, and intelligent spectral enhancement. These cutting-edge approaches enable unprecedented detection sensitivity achieving sub-ppm levels while maintaining >99% classification accuracy, with transformative applications spanning pharmaceutical quality control, environmental monitoring, and remote sensing diagnostics.

## Graphical abstract

**Figure:**
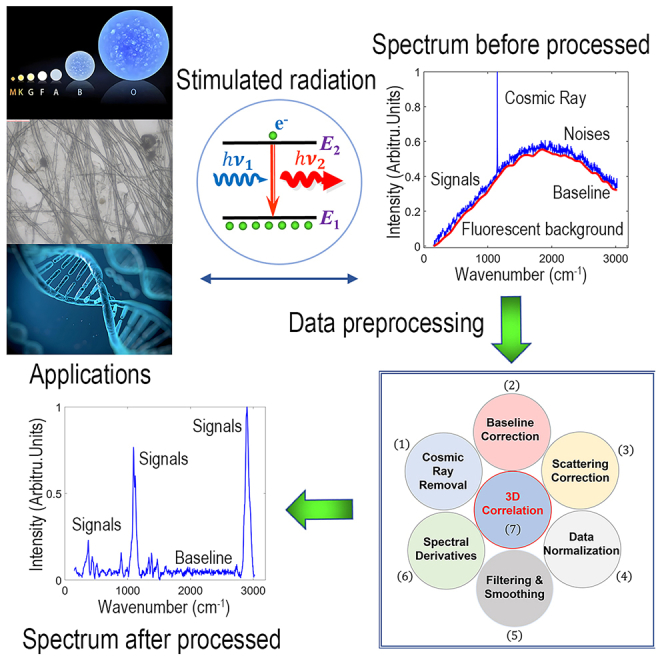

## Introduction

Spectral analysis is a fundamental tool for material characterization,^1^^,^^2^^,^^3^^,^^4^^,^^5^^,^^6^^,^^7^^,^^8^^,^^9^^,^^10^ yet its effectiveness is consistently challenged by both intrinsic signal limitations (e.g., low photon yields or peak shifts from molecular interactions) and extrinsic perturbations (e.g., environmental fluctuations inducing baseline drifts or tilts), all of which undermine quantification accuracy. For instance, in gamma-ray spectroscopy, cosmic ray artifacts obscure faint astrophysical signals^11^; in mid-infrared flame spectroscopy, thermal radiation introduces nonlinear baselines that impede gas concentration retrieval^12^; while in ocean color remote sensing, instrument noise exacerbates errors in atmospheric correction for parameters like total suspended solids (TSS).^13^ Beyond domain-specific noise, cross-instrument variability and methodological inconsistencies further amplify these distortions, impairing both traditional analyses (e.g., Beer-Lambert–based quantification) and machine learning models (principal component analysis (PCA),^14^ partial least squares (PLS),^15^ support vector machines (SVMs),^16^ k-nearest neighbors (KNN),^17^ and convolutional neural networks (CNNs)^18^). Despite the critical role of preprocessing, existing review articles and methodological discussions often focus narrowly on domain-specific applications or specialized spectral processing techniques, lacking a unified mathematical framework or broad generalizability.^19^^,^^20^^,^^21^^,^^22^^,^^23^^,^^24^^,^^25^^,^^26^

**Figure 1:**
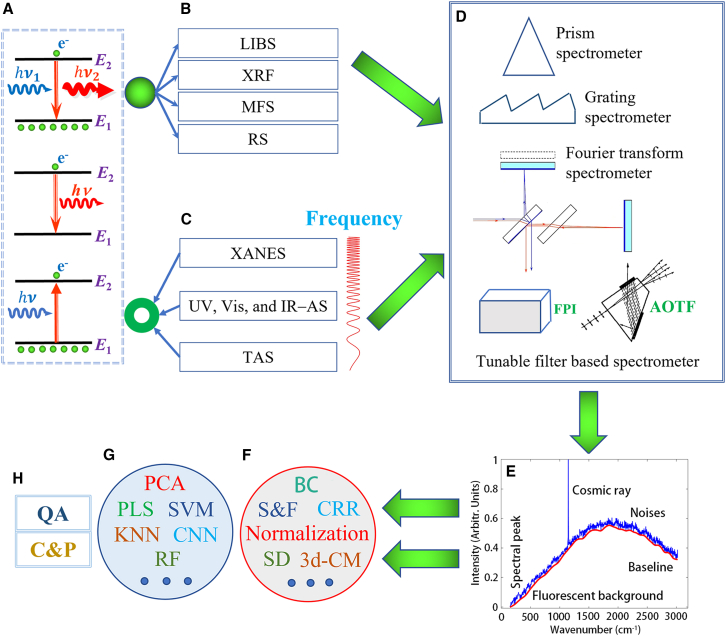
Schematic diagram of spectral technology principle The figure summarizes fundamental energy transitions (A), spectral classifications (B-C), instrumentation (D), spectral data structure and preprocessing (E and F), machine learning methods (G), and applications (H). (A) **Energy level diagrams** of spontaneous emission, stimulated emission, and stimulated absorption. (B) **Emission spectra classifications:** LIBS (laser-induced breakdown spectroscopy), XRF (X-ray fluorescence spectroscopy), MFS (molecular fluorescence spectroscopy), and RS (Raman spectroscopy). (C) **Absorption spectra classifications:** XANES (X-ray absorption near-edge structure spectroscopy), UV (ultraviolet), Vis (visible), IR (infrared), AS (absorption spectroscopy), and TAS (terahertz absorption spectroscopy). (D) **Spectrometer types:** FPI (Fabry–Perot interferometer) and AOTF (acousto-optic tunable filter). (E) **Spectral data structure with interference factors:** Noise, baseline drift, cosmic rays, and fluorescent background. (F) **Preprocessing methods:** Normalization, BC (baseline correction), S&F (smoothing and filtering), CRR (cosmic ray removal), SD (spectral derivative), and 3days-CM (three-dimensional correlation method). (G) **Machine learning methods:** PCA (principal component analysis), PLS (partial least squares), SVM (support vector machine), KNN (k-nearest neighbor), RF (random forest), and CNN (convolutional neural networks). (H) **Applications:** QA (quantitative analysis) and C&P (classification and prediction).

At the quantum level, spectroscopic signals arise from electron/phonon transitions (emission or absorption^27^^,^^28^; Figure 1A), manifesting as emission spectra (e.g., laser-induced breakdown spectroscopy (LIBS) or Raman; Figure 1B) or absorption spectra (e.g., UV-Vis or IR; Figure 1C). While absorption spectra obey the Beer-Lambert law, their practical measurement via dispersion techniques (prisms, gratings, Fourier transform interferometry, or tunable filters^29^; Figure 1D) decomposes raw signals into three components: target peaks (physicochemical information), background interference (e.g., scattering or thermal effects), and stochastic noise (e.g., detector readout errors; Figure 1E). Whether confronting cosmic ray spikes in astrophysics or fluorescence-dominated Raman spectra, these artifacts invariably mask intrinsic spectral features (Figure 1G), necessitating systematic preprocessing to recover latent material signatures.

To address these challenges, we propose a hierarchy-aware preprocessing framework (Figure 1F) comprising: (1) Localized artifact removal (cosmic ray/spike filtering), (2) baseline correction for low-frequency drift suppression, (3) scattering correction, (4) intensity normalization to mitigate systematic errors, (5) noise filtering and smoothing, (6) feature enhancement via spectral derivatives, and (7) information mining by three-dimensional correlation method. This pipeline (Figures 1H; Table 1) synergistically bridges raw spectral fidelity and downstream analytical robustness, ensuring reliable quantification and machine learning compatibility.

**Table 1:** Comparison of spectral data preprocessing techniques: mechanisms, validation requirements, and applications

Category	Method	Core Mechanism	Validation Need	Advantages	Disadvantages	Primary Role & Application Context
**Cosmic Ray Removal**	**Moving Average Filter (MAF)**^30^	Detects cosmic rays via MAD-scaled *Z* score and first-order differences; corrects with outlier rejection and windowed averaging (Equations 1 and 2).	Assumes isolated CRAs and single-scan spectral data. Threshold τ needs to be calibrated to 3–5.	Fast real-time processing with better spectral preservation than uniform averaging.	Blurs adjacent features due to uniform-weight averaging; sensitive to window size (m) tuning.	Real-time single-scan correction for Raman/IR spectra without replicate measurements.
	**Missing-Point Polynomial Filter (MPF)**^31^	Explicitly excludes central outlier (zero-weighted convolution $C_{2,0,i}=0$), fits quadratic polynomial via least squares (Equation 3).	Assumes sparse CRAs and uniform spectral sampling (e.g., Raman/IR).	Preserves fidelity by excluding corrupted points; faster than Savitzky-Golay.	Ineffective for clustered CRAs; requires uniform spectral sampling (e.g., Raman/IR).	Optimized for real-time cosmic ray removal in Raman/IR spectra (single-scan, uniform sampling), prioritizing local feature fidelity.
	**Multistage Spike Recognition (MSR)**^32^	Forward differences ($D_{\left(k,m\right)}=S_{\left(k+1,m\right)}-S_{\left(k,m\right)}$) + dynamic threshold ($t_{\left(1,j\right)}=4\sigma_{\left(1,j\right)}$). Shape validation (sharp rise/fall, width ≤30 pixels) (Equation 4).	Requires ≥40 sequential spectra. adaptive threshold performance verification.	Automated: Dynamic thresholds auto-adjust. Accurate: shape constraints ensure precision. Robust: drift resilience maintains signal integrity.	May miss broad anomalies (>30 px) due to rigid width constraints.	Reliable cosmic ray removal for time-resolved Raman spectra (40+ scans) with drift/variable spikes.
	**Polynomial Filtering (PF)**^33^	Global third-order (or higher) polynomial fit per wavenumber; outliers rejected at > 3σ noise deviation.	Requires manual validation or median filtering for heterogeneous data (avoids distortion).	Spectral-preserving, Robust automation, Scalable for high-throughput (homogeneous data).	Struggles with local anomalies in heterogeneous data. requires manual validation.	Optimized for high-throughput Raman applications where manual inspection is impractical.
	**Nearest Neighbor Comparison (NNC)**^34^^,^^35^	Normalized covariance similarity ($C_{nm}$) + Savitzky-Golay noise estimation ($\sigma_{n}$); dual thresholds ($5\sigma_{n}$ primary, $2\sigma_{n}$ secondary) (Equations 5 and 6).	Requires validation of single-scan robustness, dual-threshold efficacy, preprocessing dependence, and computational speed.	Single-scan avoids read noise; auto-dual thresholds optimize sensitivity/specificity.	Assumes spectral similarity; SG smoothing affects low-SNR regions.	Real-time hyperspectral imaging or time-sensitive spectroscopic analysis under low SNR conditions.
	**Wavelet Transform (DWT+K-means)**^36^^,^^37^^,^^38^	DWT decomposition (depth $n\approx \log_{2}\left(N\right)-3$) + K-means clustering; Allan deviation threshold (3×ADEV) (Equations 7 and 8).	Requires testing on datasets with CR/Raman FWHM overlap.	Multi-scale wavelet analysis preserves spectral details; automated for large datasets (single-scan).	Limited efficacy when CRA width overlaps Raman peaks (low-resolution data).	Single-scan CRA correction for Raman spectra (high-frequency artifacts).
	**Kernel PCA Residual Diagnosis (KPCARD)**^39^^,^^40^	Gaussian KPCA ($K_{ij}$) + dual-threshold residual diagnosis ($t_{1}$, $t_{2}$); reconstruction via PCA components (Equations 9, 10, 11, and 12).	Requires clear contrast between artifacts and true spectra	KPCA preserves subtle features, robust to noise, retains true spectral profiles.	Computationally intensive; requires manual optimization of σ and PCA order.	High-precision artifact removal for nonlinearly distorted spectra (e.g., high-resolution Raman).
**Baseline Correction**	**Wavelength Artificial Shift Subtraction (WASS)**^41^	Artificial wavelength shift (Δλ) isolates signal via least-squares solution. Shift magnitude must avoid noise amplification (Δλ too large) or insufficient separation (Δλ too small). (Equations 14, 15, 16, and 17).	Requires precise Δλ calibration (avoid under/over-shifting).	No empirical fitting. Robust for obscured signals (e.g., underwater LIBS).	Suboptimal Δλ degrades performance; sensitive to shift magnitude.	Underwater LIBS: Targets faint emission lines and Resists obscuration & noise.
	**Piecewise Polynomial Fitting (PPF)**^42^^,^^43^^,^^44^^,^^45^^,^^46^^,^^47^	Segmented polynomial fitting (orders adaptively optimized per segment) with S-ModPoly variant for iterative refinement (Equation 18).	Poor segmentation in PPF leads to over/underfitting → adaptive methods (e.g., S-ModPoly) needed for robust baselines.	Adaptive & Fast: No physical assumptions, handles complex baselines. Rapid (<20 ms, Raman) with smooth continuity (S-ModPoly).	Sensitive to segment boundaries and polynomial degree (over/underfitting). Requires adaptive methods (e.g., S-ModPoly) for robustness.	High-accuracy soil analysis: 97.4% land-use classification via chromatography.
	**B-Spline Fitting (BSF)**^48^^,^^49^^,^^50^^,^^51^	Local polynomial control via knots (T) and recursive basis ($B_{j,k}$). Least-squares optimizes control points (Equations 19, 20, 21, and 22).	Tune knots & degree (k)—accuracy drops if suboptimal.	Local control avoids overfitting, boosting sensitivity 3.7× for gases (NH_3_/O_3_/CO_2_).	Scales poorly with large datasets unless optimized; knot degree tuning critical.	Robust trace gas analysis—resolves overlapping peaks & irregular baselines.
	**Two-Side Exponential (ATEB)**^52^	Bidirectional exponential smoothing (forward/backward passes) with adaptive weights α (Equation 23).	Tunable smoothing: Optimize α (smoothing) and ε (convergence) for balance between noise reduction and signal fidelity.	Fast & automatic: Linear O(n) time, scalable for large data. Self-adjusting—no manual peak tuning.	Less effective for sharp fluctuations (B-splines preferred).	Best for: High-throughput data with smooth/moderate baselines.
	**Morphological Operations (MOM)**^53^^,^^54^^,^^55^^,^^56^^,^^57^^,^^58^^,^^59^	Erosion/dilation with structural element (width 2l+1). Averaged opening/closing. Mollifier convolution (Equations 24, 25, 26, and 27).	Structural element width (2l+1) must match peak/trough dimensions; RCR threshold controls adaptivity.	Key Benefits: Maintains spectral peaks/troughs (geometric integrity) and Optimized for Pharma PCA workflows (classification-ready).	Trade-offs: ① SE too wide: Blurs sharp peaks; ② SE too narrow: Underfits baseline; and ③ Accuracy depends on window alignment.	Pharmaceutical tablet analysis, enhancing peak-valley distinction while suppressing noise.
	**Singular Value Decomposition (SVD)**^59^^,^^60^^,^^61^	Rank-k SVD decomposition ($Y=U\sum V^{T}$) isolates baseline (noise suppressed); residual enhances features (Sharp peaks/valleys) (Equations 28, 29, and 30).	Optimal Rank-k Selection: via Variance threshold or Fuzzy GSVD.	Preserves signal features (e.g., boosts $R^{2}$ from 0.55 to 0.88). Fast for large datasets (GC-TOF-MS, NIR).	Performance sensitive to k selection (misestimation impacts correction).	Automated baseline correction (NIR, DPPH/ABTS) with high accuracy.
	**Wavelet Transforms (DWT)**^62^^,^^63^^,^^64^^,^^65^^,^^66^^,^^67^^,^^68^	DWT decomposes signals into low-frequency baselines ($A_{J}$) and high-frequency details ($D_{J}$), using filter banks ([h]/[g]) for exact reconstruction (Mallat) (Equations 31 and 32).	Requires wavelet choice (e.g., db4) and thresholding for artifact suppression.	No shape assumptions—localized analysis with strong noise resistance (e.g., 21× lower RMSE vs. polynomial fitting).	Potential minor artifacts (e.g., negative lobes) from wavelet oscillations.	Terahertz & complex spectra—precise baseline/peak separation.
	**Least Squares Methods (LSM)**^69^^,^^70^^,^^71^^,^^72^^,^^73^^,^^74^^,^^75^^,^^76^^,^^77^^,^^78^^,^^79^^,^^80^^,^^81^^,^^82^^,^^83^^,^^84^^,^^85^	**Weighted Penalized Least Squares (wPLS):** Combines weighted residuals (fidelity F) and curvature smoothing (roughness penalty R via 2nd-order differences) (Equations 33, 34, 35, and 36).	Requires λ tuning (L-curve/cross-validation).	Explicit baseline modeling, robust to drift-dominated noise (Raman/IR).	Limited flexibility for mixed-frequency artifacts.	Raman/IR spectroscopy with smooth baselines.
		**Asymmetric Least Squares (AsLS):** Applies asymmetric weights ($\omega_{i}^{\text{AsLS}}$) to penalize peaks while fitting smooth baselines via second-order difference regularization ($D_{2}$) in least squares optimization. (Equation 37).	Tune λ via L-curve; validate against peak suppression in noisy regions.	Effective for gentle baselines (e.g., weak Raman drift). Preserves peaks via asymmetric penalties.	Oversmooths weak peaks near baseline. Struggles with mixed-frequency artifacts (jumps + drift).	Baseline correction for spectra with smooth/moderate drift and distinct peaks (e.g., Raman, weak signals).
		**Improved AsLS (IAsLS):** extends AsLS by incorporating dual regularization alongside asymmetric weights to address mixed-frequency baseline artifacts. (Equations 37 and 38).	Empirical trade-off between $\lambda_{1}$ (jump suppression) and $\lambda_{2}$ (curvature control) via cross-validation.	Handles mixed artifacts (e.g., NMR drift + Raman jumps). Adaptive to both sharp and smooth distortions.	Requires manual tuning of $\lambda_{1}/\lambda_{2}$. Computationally heavier than AsLS.	Complex baselines with combined distortions (e.g., IR spectra with abrupt baseline shifts + gradual drift).
		**Multiple Constrained AsLS (mcaLS):** Enforces peak symmetry via left/right residual equalization (Equation 39).	Optimize $\lambda_{1}$/ $\lambda_{2}$ to balance peak symmetry and smoothness.	Improved RMSE (e.g., 0.09 vs. exponential baselines).	Computationally intensive.	NIR spectra with complex baselines (e.g., maize).
		**Adaptive Iteratively Reweighted PLS (airPLS) & Improved airPLS (IairPLS):** Exclude peaks ($w_{i}^{t}=0$) and fit baselines via adaptive weights (exponential in airPLS, sigmoid in IairPLS), balancing residuals and smoothness with penalty term λ (Equations 40, 41, and 42).	Requires noisy-data validation (e.g., XRF) for baseline RMSE and peak-interference robustness.	airPLS: Simple, fast, effective for mild noise. IairPLS: Higher accuracy (e.g., RMSE 0.0187 vs. 0.0531 in XRF), fewer edge artifacts.	airPLS: Underestimates noisy baselines; λ-sensitive. IairPLS: Slower; needs sigmoid tuning.	Smart baseline fitting for spectra/chromatograms (XRF/Raman/HPLC).
		**Asymmetrically Reweighted PLS (arPLS):** Logistic sigmoid weighting ($w_{i}^{t}$) dynamically suppresses negative residuals (noise) while preserving peaks (Equation 43).	Tune λ (smoothness) via L-curve; validate against false peak suppression in noisy regions.	Automated noise adaptation. Effective for Gaussian/symmetric noise (e.g., XRF).	Overfits faint peaks near baseline.	General-purpose correction for medium-SNR spectra (e.g., XRF, NIR, UV-Vis) with smooth baselines, symmetrical noise, and moderate drift.
		**Inverse Square Root Unit (ISRU):** Inverse square root ($w_{i}^{t}=\text{ISRU}\left(d_{i}\right)$) smooths transitions, better capturing weak peaks. (Equation 44).	Validate trade-off between peak sharpness and noise robustness in baseline correction.	Superior weak-peak retention.	N/A	N/A
		**Sparse Bayesian Learning (SBL/BrPLS):** Bayesian weight adaptation ($w_{i}$) to separate baseline ($z_{i}$) and signal, using noise (σ) and amplitude (μ)parameters (Equations 45 and 46).	N/A	Avoids heuristic tuning (adaptive β,σ,μ). Rigorous $l_{0}$-norm minimization via Bayesian inference. Outperforms arPLS/airPLS/AsSL in noise resilience and accuracy.	Computationally heavy (Bayesian updates). Needs prior β estimation (signal probability).	Probabilistic baseline correction for noisy, overlapping-peak spectra, excelling in accuracy and robustness over traditional methods.
	**Sparse Representation Method (SRM)**^86^^,^^87^^,^^88^^,^^89^^,^^90^	**Simultaneous Spectrum Fitting and Baseline Correction via Sparse Representation (SSFBCSP):** Sparsity-based joint fitting of baseline and peaks with smoothness/sparsity constraints (Equations 47 and 48).	Requires rigorous validation for highly overlapping peaks (e.g., Raman) and irregular baselines (e.g., fluorescence/drift).	High precision (RMSE 7.83 × 10^−4^). Adaptable dictionaries handle complex peak shapes.	Slow (dictionary learning); sensitive to prior knowledge in dictionary selection.	Gold-standard for baseline/peak separation. Ideal for biomedical spectroscopy, Raman/NIR, and complex spectral data.
		**Fast Burst-Sparse Learning for Baseline Correction (FBSL-BC):** Fast baseline correction via downsampling and adaptive sparse coding (Voigt dictionary), optimized for burst-sparse signals (Equation 49).	Requires high-throughput validation for real-time processing of large datasets (e.g., agricultural/industrial spectrometry).	3–5× faster than SSFBCSP. Balanced accuracy-speed trade-off (e.g., $R^{2}=0.9647$ on corn NIR dataset).	Lower accuracy with weak signals; struggles with irregular baselines.	Fast, scalable solution for portable spectrometers, agri-NIR, and industrial monitoring.
	**Convex Optimization (COF)**^91^^,^^92^^,^^93^^,^^94^	Unified high-pass fidelity ($H=BA^{-1}$),adaptive sparsity ($\theta_{\varepsilon }$), and smoothness($D_{i}$), via MM optimization Equations 50, 51, 52, 53, and 54.	N/A	Automation: Reduces manual tuning. Feature retention: Preserves critical peaks (Raman/LIBS) while removing baselines. Adaptivity: Dynamic sparsity-smoothness trade-off via $\theta_{\varepsilon }$.	Complex parameter tuning ($\lambda_{i}$, ε, r); requires inversion $A^{-1}$.	Spectral signal enhancement, especially for LIBS/Raman with baseline drift and overlapping peaks (e.g., steel composition analysis).
	**Bézier Curve Fitting**^87^^,^^95^^,^^96^	Parametric curve defined by control points $b_{i}$ and Bernstein polynomials $B_{i}^{n}\left(t\right)$ , satisfying convex hull containment, endpoint interpolation, and tangent alignment (Equation 55).	N/A	Stable (strict convexity) and simple (endpoint interpolation).	No local control (less flexible than B-splines); constrained to global convexity.	Convex spectral fitting: prioritizes stability over flexibility.
	**Genetic Algorithm (GA)**^97^^,^^98^^,^^99^^,^^100^^,^^101^^,^^102^^,^^103^	Evolutionary optimization of fitness function balancing peak retention ($A_{p}$) and baseline distortion ($A_{z}$) under noise constraints (Equation 56).	N/A	Self-optimizing automated baseline correction: dynamically balances peak preservation and artifact removal with noise-aware precision.	Computationally demanding for large datasets or high-generation optimizations.	Automated high-throughput spectral analysis (Raman/clinical/agricultural) outperforms manual methods (e.g., Bézier) in precision, robustness, and efficiency.
	**Deep Learning (1D-CNN/GAN/VAE)**^104^^,^^105^^,^^106^^,^^107^^,^^108^^,^^109^^,^^110^^,^^111^^,^^112^	**1D Convolutional Neural Networks (1D-CNNs):** ReLU-activated convolutions with MSE optimization (Equations 57 and 58).	N/A	Automated, high-accuracy feature extraction (95.09% in MIR breast cancer classification).	Poor interpretability of learned filters. Requires large datasets for kernel optimization.	MIR spectroscopy for biomedical classification.
		**Enhanced GAN (EGAN):** Adversarial training with generator (G) and LSGAN discriminator (D); composite loss includes $l_{1}$ reconstruction and local peak preservation (Equations 59, 60, and 61).	N/A	Handles high variability ($R^{2}>0.99$ for XRF alloys) with transferability to new domains (e.g., soils).	Dual-network training is computationally intensive and sensitive to β.	Robust XRF spectroscopy for alloys and soils, handling drifting baselines.
		**Variational Autoencoders (VAEs):** Gaussian latent space ($\mu_{z}$, $\sigma_{z}^{2}$); optimizes ELBO (reconstruction + KL loss) (Equations 62, 63, 64, 65, and 66).	N/A	Uncertainty-aware VAE: Reconstructed ( ${\hat{u}}_{x^{\prime }}$, ${\hat{\sigma }}_{x^{\prime }}^{2}$) with edge deployment (TinyML-VAE NRMSE: 0.53–0.66).	VAE issues: Over-smoothing, parameter sensitivity.	Error-Sensitive Spectroscopy in Embedded Sensing (e.g., Magnetometers).
		**Enhanced U-Net & Hybrid Models:** Learned Feature Fusion (LFF) + Residual Dense Blocks (RDBs). Three-stage training: unpaired pretraining, synthetic augmentation, contrastive fine-tuning.	N/A	Near-perfect regression ($R^{2}\approx 0.99$), preserving features without over-smoothing.	N/A	High-order baseline correction (7th-degree poly) for Raman/seismic spectra.
**Scattering Correction**	**Multiplicative Scatter Correction (MSC)**^113^^,^^114^^,^^115^^,^^116^	Linear correction of additive ($c_{i}$), & multiplicative ($b_{i}$) effects via OLS regression against reference spectrum ${\bar{x}}_{k}$ (Equations 67, 68, 69, 70, and 71).	Validate linearity (e.g., spectral homogeneity) on representative data.	Robustly corrects additive & multiplicative effects (e.g., baseline shifts, path-length). Preserves chemical features (e.g., FTIR oil analysis: $R^{2}=0.944$).	Nonlinear limitation: Ineffective for scattering (e.g., complex biological tissues).	Empirical pre-processing for scatter-prone FTIR data (e.g., oil/moisture analysis).
	**Extended MSC (EMSC)**^117^^,^^118^^,^^119^^,^^120^^,^^121^	Extends MSC with polynomial ($\sum d_{i}{\tilde{\nu }}^{i}$) and PCA terms ($\sum g_{k}p_{k}\left(\tilde{\nu }\right)$) for interferents & nonlinear baselines (Equations 72, 73, and 74).	Cross-validation selects polynomial order (n) and PCA components to avoid overfitting.	Superior to MSC for complex samples (e.g., tissues, multicomponent systems). High specificity (e.g., 97% for Crohn’s). Reduces artifacts (e.g., temperature effects).	Requires careful tuning. Computationally heavier than MSC due to extra terms and PCA. Requires >95% cumulative variance in PCA.	Advanced spectral correction for complex signals (nonlinearities, noise, interference). Used in biomedical (FTIR), polymers, and systems with confounders (e.g., moisture, temperature).
	**Detrending (DT)**^122^^,^^123^	Subtracts least-squares-fitted polynomial $B\left(\lambda \right)=\sum a_{i}\lambda^{i}$ from y(λ) to eliminate drift (Equation 76).	Requires optimizing polynomial order (N) to avoid overfitting while removing drift.	Simple, effective for low-frequency drift (e.g., 97.4% TCM species ID, enhanced NIRS accuracy).	Risk of overfitting; baseline-only correction (needs pairing, e.g., SNV).	Corrects baseline drift via polynomial fitting, often paired with SNV (e.g., NIRS nutrient prediction) and Traditional Chinese Medicine spectral analysis.
	**Standard Normal Variate (SNV)**^124^^,^^125^^,^^126^	Normalizes spectra (mean = 0, variance = 1) to reduce scatter/amplitude effects (Equation 77).	Assess risk of feature suppression (e.g., geometric influences).	Robust sample-wise processing handles heterogeneity (scattering/baselines). Highly effective (e.g., 99% ginseng authentication accuracy).	Excessive scaling may suppress useful variance (geometric methods can outperform). Limited to single-spectrum normalization.	Optimized for high-frequency, sample-specific noise (e.g., scattering, NIRS, LIBS) in amplitude-driven data.
**Data Normalization**	**Mean Centering (MCN)**^127^^,^^128^^,^^129^^,^^130^^,^^131^	Subtracts arithmetic mean $\bar{x}$ from each $x_{i}$ to center data (Equation 78).	Preserves discriminative features while removing offsets.	Removes instrumental drift, preserves spectral integrity—critical for PCA (e.g., 99.7% NIR accuracy).	Fixes global shifts, not local/multiplicative effects.	Key for PCA/PLS baseline alignment—vital in agriculture and pharmaceuticals analysis.
	**Orthogonal Signal Correction (OSC)**^132^^,^^133^^,^^134^^,^^135^^,^^136^^,^^137^^,^^138^^,^^139^^,^^140^^,^^141^	Removes Y-orthogonal noise via PCA/SVD projections of residual matrix Z (Equations 79, 80, 81, 82, 83, 84, and 85).	Targeted non-correlated noise removal, preserves predictive signals.	Boosts robustness via structured noise removal (e.g., rice bran oil analysis: $R^{2}:0.50\to 0.987$).	Higher compute cost vs. univariate methods.	Applies to spectroscopy noise reduction (e.g., oil analysis, glucose monitoring, soil measurement).
	**Min-Max Normalization (MMN)**^127^^,^^142^^,^^143^^,^^144^^,^^145^^,^^146^^,^^147^	Normalizes data to [0,1] via min-max scaling (Equation 86).	N/A	Simple, universally beneficial, and performance-enhancing. Universal scaling for fixed-range models (e.g., honey classification: 97%).	Highly sensitive to outliers (min/max-dependent).	Applied in spectroscopy, environmental analysis, and biomedical models for generic feature scaling.
	***Z* Score Normalization (ZSN)**^148^^,^^149^^,^^150^^,^^151^^,^^152^	Standardizes data to zero mean and unit variance. Standardizes to u=0, σ=1 via $z_{i}=\left(x_{i}-\bar{x}\right)/\sigma$ (Equations 87 and 88).	Verify ① $\bar{z}\approx 0$ and $\sigma_{z}\approx 1$, ② outlier robustness (vs. min-max), and ③cross-domain consistency (e.g., radiomics harmonization).	Robust to outliers (vs. MMN), suppresses noise (reduces multicollinearity), and adapts across domains (supports LSNV variants).	Assumes Gaussianity (non-normal distortion risk). Global scaling (may disrupt local patterns).	Applied in spectroscopy (artifact removal, classification), medical imaging (cross-scanner harmonization), high-dimensional data (multicollinearity reduction).
	**3-Sigma Normalization (3-SN)**^127^^,^^153^	Truncates outliers beyond $\bar{x}\pm 3\sigma$, scales inliers to [0,1] via $z_{i}=\left[x_{i}-\left(\bar{x}-3\sigma \right)\right]/6\sigma$ (Equation 89).	Truncation Bounds: Confirm $\bar{x}\pm 3\sigma$ (3σ threshold). Normalization: Verify scaled values ∈[0, 1] (post-truncation).	Outlier robustness (vs. *Z* score). Noise filtering. High classification accuracy (97.8% in breast tumors). Cross-scanner stability.	Information loss (eliminates extreme values). Less theoretical foundations (heuristic approach).	Robust spectroscopic tumor classification method: Cross-scanner harmonization + outlier robustness + signal fidelity.
	**L1 Normalization**^154^	scales feature by Manhattan norm (sum of absolute values) to preserve sparsity (Equation 90).	Verify $\sum \left|x_{j}\right|$ normalization preserves sparsity (e.g., NILM accuracy: 96.42–97.65%).	Sparsity retention (key for NILM). Preserves appliance signatures (96.42–97.65% accuracy, PLAID/WHITED).	Sensitive to low-magnitude noise (non-zero values may be amplified). Ignores distributional properties (vs. ZSN/3-SN).	Non-intrusive load monitoring (NILM) via sparse signal decomposition (e.g., energy disaggregation).
	**L2 (Vector) Normalization**^155^^,^^156^	Scales by Euclidean norm: $z_{i}=x_{i}/{\left\|x\right\|}_{2}$ (Equation 91).	Ensure $l_{2}$ normalization enhances discrimination without distorting angular relationships.	Preserves cosine similarity. Enhances contrast (100% PPCA accuracy). Compacts data (88.6% lossless spectral reduction).	Ignores magnitude (angle-focused). Noise-sensitive (non-sparse-optimized).	Angular similarity tasks (e.g., cosine distance). Dimensionality reduction (e.g., VN-CARS-PLSR for kiwifruit sugar prediction). Direction-sensitive classification (e.g., brain MR analysis).
	**Logarithmic Transformation (LT)**^157^^,^^158^	Converts nonlinear (multiplicative) relationships into additive space $z_{i}=\log_{b}\left(x_{i}+c\right)$ (Equation 92).	Ensure input stability (requires $x_{i}+c>0$)	Stabilizes variance, normalizes skewness, and compresses dynamic ranges (e.g., RMSEP = 128 in pulp quality analysis).	Excludes non-positive data (requires $x_{i}+c>0$). Less flexible than alternatives (e.g., Box-Cox for power-law data).	Spectral/exponential data (NIR absorbance, soil carbon prediction). Variance stabilization for skewed distributions.
	**1/n Power Transformation (1/n-PT)**^159^	Nonlinearly compresses values with parametric control: $z_{i}=x_{i}^{\frac{1}{n}}$ (Equation 93).	Ensure non-negative inputs (requires $x_{i}\geq 0$); shift data if negative ($x_{i}+c$).Hyperparameter tuning for optimal n.	Reduces outliers, normalizes skewness, stabilizes variance, and optimizes feature distributions.	Limited to non-negative data (requires shifting for negatives). Compression efficacy depends on n, requiring careful tuning.	Spectroscopic analysis (e.g., Cu(II)-DOM binding quantification, humic substance enrichment). Parametric compression for skewed data (e.g., TOC removal: 89%).
**Filtering & Smoothing**	**Gaussian Filtering (GF)**^160^^,^^161^	Convolves spectra with Gaussian kernel $G\left(x\right)=e^{-\left(x^{2}/2\sigma^{2}\right)}/\sqrt{2\pi \sigma^{2}}$. Cutoff frequency $f_{c}\approx 1/\left(2\pi \sigma \right)$ (Equation 94).	Optimal σ tuning: Avoid under-smoothing (residual noise) or over-smoothing (peak distortion).	Reduces high-frequency noise while retaining spectral features. Adaptive cutoff: $f_{c}\approx 1/\left(2\pi \sigma \right)$.	Balancing noise suppression and peak distortion requires careful tuning of σ.	LIBS preprocessing: Resolves emission lines (e.g., Li I 670.8 nm). Enables 100% accurate Dendrobium species classification.
	**Savitzky-Golay Filter (SGF)**^162^^,^^163^^,^^164^^,^^165^^,^^166^^,^^167^^,^^168^	Sliding-window local polynomial fitting (size 2M+1): smoothed output p(k) corresponds to the central fitted value (Equation 95).	Optimal tuning of window size (M) and polynomial degree (N).	Preserves spectral moments up to order N (unlike fixed GF). Improves SNR (e.g., 2.35× in methane detection) without peak distortion.	Polynomial degree N overfits noise if too high; window size M blurs features if too large.	Methane detection (LITES), CO monitoring (Vis-NIR), soil phosphorus prediction via remote sensing ($R^{2}=0.81$), and gas analysis (TDLAS, 0.53 ppm accuracy).
	**Wiener Filtering (WF)**^169^^,^^170^^,^^171^^,^^172^	optimally estimates signals by adaptively weighting observations based on noise and system response in the frequency domain (Equations 96 and 97).	Requires accurate $P_{ss}\left(\nu \right)$ and $P_{nn}\left(\nu \right)$ estimation.	Superior noise suppression (vs. SGF in SNR-adaptive cases). 100 ppm CH_4_ detection limit (vs. SGF’s 120 ppm). 40% higher resolution (vs. SGF).	Requires accurate $P_{ss}\left(\nu \right)$ (signal power spectrum) and and $P_{nn}\left(\nu \right)$ (noise power spectrum) estimation.	Precision spectroscopy: CH_4_ detection, hyperspectral imaging. Miniature spectrometers: Better abrupt noise suppression vs. SGF. Motion artifact removal: NIR spectroscopy.
	**Kalman Filtering (KF)**^173^^,^^174^^,^^175^^,^^176^^,^^177^^,^^178^^,^^179^	Recursive estimation: Time update (model prediction) + measurement update (sensor data). Optimal weighting: Kalman gain $K_{k}$ minimizes error covariance $P_{k}$ (Equations 98, 99, and 100).	Verify A, H, Q, R accuracy; recursive updates minimize posterior error covariance $P_{k}^{-}$.	Minimizes MMSE via Kalman gain $K_{k}$, reduces NO_2_ detection limits 9.12×, accelerating isotope analysis (CO_2_ δ^13^C/δ^18^O) 7-fold, and cutting PLS RMSE by 40% (0.38 → 0.17 g/L).	Model sensitivity: Performance affected by inaccurate A, H, Q, R. Computational load: Higher than static methods (e.g., WF) due to recursive updates.	NO_2_ cavity ring-down (transient noise suppression). Glucose Raman (real-time noise reduction). LIBS steel analysis (drift correction). QCL isotopologues (high-speed precision).
	**Neural Network Adaptive Filter (NNAF)**^180^^,^^181^^,^^182^	LMS weight updates with sigmoid-step control: $u=\left[-0.5+1/\left(1+e^{-a\left|x\right|}\right)\right]\left(e^{{\left|x\right|}^{r}}-1\right)$ (Equations 101, 102, 103, and 104)	Requires tuning of Step-size bounds ($u_{\max}$, $u_{\min}$), Sigmoid steepness (a), and Error exponent (r). Must satisfy $\beta <n/\lambda_{\max}$ for convergence.	Higher adaptability in nonlinear environments; 3–4 dB SNR gain (UV-Vis) and $R^{2}>0.99$ f for concentration prediction.	Sensitive to a, r, and step-size. Higher computational cost than LMS/KF.	Spectroscopic denoising (Zn extraction, Cu/Co impurity analysis). Nonlinear signal processing (beyond KF/WF assumptions).
	**Wavelet Shrinkage Denoising (WSD)**^183^^,^^184^^,^^185^^,^^186^	Applies universal threshold $\lambda =\sigma_{j}^{Noise}\sqrt{2\;\ln\;N}$ or level-dependent $\lambda_{j}=\sigma_{j}^{Noise}\sqrt{2\;\log_{2}\left(N_{j}\right)}$ to wavelet coefficients. (Equations 105, 106, 107, 108, 109, and 110).	Requires: Wavelet basis selection (e.g., Daubechies), Optimal decomposition level ($k=\left[\log_{2}\;N\right]$), Noise estimation (e.g., MAD). Threshold tuning (noise removal vs. signal fidelity)	Employs MAD-based noise estimation, reducing GFRP thickness error to 3.7% (↓16.4%→3.7% via THz-TDS) with fast sparse processing; achieves high noise reduction (+32 dB SNR gain in ESR spectroscopy).	Basis dependency (sensitivity to wavelet choice). Transient distortion (potential oversmoothing of sharp features).	GFRP thickness analysis. ESR/THz-TDS signal enhancement.
	**Hilbert Vibration Decomposition (HVD)**^187^^,^^188^^,^^189^^,^^190^^,^^191^	Adaptive demodulation (Hilbert-based component extraction). Iterative decomposition (dominant component and residual processing). Stopping criterion (Iterates until frequency estimate SD δ≤0.001) (Equations 111, 112, 113, 114, and 115).	Requires Reference frequency ($\omega_{r}$) selection, Convergence threshold (δ) tuning, and AM/FM signal suitability verification.	SNR improvement: 3.53 → 130.64 (spectroscopy). Classification accuracy: 40% → 90.25%.	Limited to AM/FM signals; sensitive to reference frequency $\omega_{r}$ selection.	Structural vibration analysis. Spectroscopic signal enhancement.
**Spectral Derivatives**	**Finite Difference Method (FDM)**^22^	Discrete differentiation: Approximates derivatives using adjacent points ($y_{i}$, $y_{i-1}$, $y_{i+1}$). First derivative ($y_{i}^{\prime }$,error O(h)) and Second derivative ($y_{i}^{\prime \prime }$,error $O\left(h^{2}\right)$) (Equation 116).	Requires Constant sampling interval (h) and Low-noise input data to avoid artifact amplification.	Computational efficiency: Simple, non-iterative calculations. Suitable for smooth, high-resolution spectra.	Noise sensitivity: Amplifies fluctuations. Truncation error: Accuracy limited by step size (h).	Primarily useful for clean, high-resolution spectra where computational simplicity is prioritized over robustness.
	**Savitzky-Golay Derivatives (SGD)**^192^^,^^193^^,^^194^^,^^195^^,^^196^^,^^197^	Polynomial fitting over sliding windows for smoothing + derivatives. Least-squares fitting. Convolution-based: Computes smoothed values and derivatives via predefined coefficient sets (e.g., $p_{s}^{\left(0\right)}$, $p_{s}^{\left(1\right)}$, $p_{s}^{\left(2\right)}$) (Equations 117, 118, 119, 120, 121, and 122).	Requires tuning Window size (n, typically ≤25) and Polynomial order (p, usually 2ND or 3rd).	Outperforming FDM in noise robustness while preserving spectral features (e.g., enabling 100% SVM accuracy in NIR wood ID), with tunable parameters (window size n and polynomial order p) for optimal smoothing-feature resolution.	Require careful selection of n and p to avoid parameter sensitivity and edge artifacts, necessitating dataset-specific optimization to maintain accuracy, particularly near spectral boundaries.	Spectral preprocessing: Baseline correction (competitive $R^{2}$ vs. NW derivatives), Real-time crystallization monitoring (2ND derivatives). Classification tasks: Paired with ML (e.g., SVM).
	**Norris-Williams Derivatives (NWD)**^198^^,^^199^	Smoothing: moving-average filter (2m+1-points window) for ${\bar{y}}_{i}$. First derivative: $\left({\bar{y}}_{i}-{\bar{y}}_{i-1}\right)/h$, Second derivative: $\left({\bar{y}}_{i+1}-2{\bar{y}}_{i}+{\bar{y}}_{i-1}\right)/h^{2}$ derivative (Equation 123).	Requires optimized m (smoothing window) and h (gap size).	Strong noise suppression, high SNR. Fast computation (real-time capable). Superior trace detection (e.g., enzyme granules, segregation index 0.71).	Potential blurring of fine features vs. polynomial methods (e.g., SGD). Parameter sensitivity: Suboptimal m/ h degrades performance.	Superior for trace component detection in particulate systems (e.g., enzyme placebo granules). Real-time particulate system monitoring (balances noise suppression & speed). Outperforms derivatives & SNV in low-SNR conditions.
	**Grünwald-Letnikov Fractional-Order Derivative (GL-FOD)**^62^^,^^200^^,^^201^^,^^202^^,^^203^^,^^204^	Employs an n×n fractional differential matrix $D^{\alpha }$ with gamma-weighted finite difference coefficients $d_{k}$ derived from Γ(z) , implemented as a sparse lower-triangular matrix truncated at k=20 for computational efficiency) (Equations 124, 125, and 126).	Optimize order α to Balance resolution and noise sensitivity. Tune step size h and truncation limit k. Examples: 0.3-order for chlorophyll, 0.6-order for soil organic matter	Captures nonlinear features better than integer derivatives (+17% correlation in some cases). Validated on hyperspectral (crops, soil, oil) and chemometric models.	Higher computational cost (matrix ops, gamma functions). Requires empirical tuning of α.	Agriculture: Cadmium detection ($R^{2}=0.84$), chlorophyll estimation ($R^{2}=0.86$). Geoscience: Soil organic matter prediction ($R^{2}=0.66$), reservoir volatility modeling. Strengths: Superior for nonlinear, low-contrast spectral features where integer derivatives fail.
**Data Information Mining**	**Three-Dimensional Correlation Method (3dCM)**^205^^,^^206^	Uses sequential Hilbert transforms (HT) and tensor products to convert 1D spectra into 3D correlation matrices, Final output has $w^{\prime \prime }=w^{3}$ equivalent resolution and augmented sample size (Equations 127, 128, 129, 130, and 131).	Confirm performance gains (<10% → >99% accuracy) in models like PCA-LR, PLS-LR, KNN, RF, CNN.	Captures latent correlations missed by removal-based methods. Significantly enhances ML performance (e.g., paper authentication).	Computationally heavy due to 3D tensor operations. Requires sufficient samples per class ($s_{k}$) for stable tensor construction.	Authentication: Handwritten paper analysis (>99% accuracy with ML). Hyperspectral: Extends 2D correlation for deeper feature extraction.

The table systematically evaluates seven key dimensions of common spectral preprocessing methods, emphasizing their theoretical foundations, practical trade-offs, and domain-specific applicability.**Columns Description:1. Category:** Broad classification of preprocessing methods, encompassing cosmic ray removal, baseline correction, scattering correction, normalization, filtering and smoothing, spectral derivatives, and data information mining.**2. Method:** Names of specific techniques within each category (e.g., Savitzky–Golay smoothing, multiplicative scatter correction).**3. Core Mechanism:** Fundamental principle or mathematical operation (e.g., Least-squares fitting, adaptive sparse coding).**4. Validation Need:** Empirical validation requirement (Yes/No) with typical approaches (e.g., noisy-data validation, manual validation).**5. Advantages:** Key strengths of each method (e.g., preserves spectral details, computationally efficient).**6. Disadvantages:** Limitations and trade-offs (e.g., computationally intensive, poor interpretability of learned filters).**7. Primary Role & Application Context:** Dominant use cases (e.g., robust XRF spectroscopy for alloys and soils).

## The removal of cosmic ray artifacts

Building upon the spectral data challenges, we focus on cosmic ray spikes (CRSs)—among the most abrupt and localized artifacts—whose immediate removal is essential to avoid propagating errors through downstream preprocessing. CRSs arise from high-energy particle collisions, manifesting as stochastic, ultra-narrowband (<1-2 cm^−1^) unipolar spikes that disproportionately distort spectral features despite their sparse occurrence. Unlike broadband noise, CRSs induce catastrophic localized distortions, corrupting both qualitative spectral interpretation (e.g., peak misidentification) and quantitative metrics (e.g., integrated intensity errors).

Convolutional smoothing (e.g., Savitzky-Golay filters) inherently fails for CRSs: their global operation sacrifices either spike suppression or genuine feature preservation—a trade-off untenable for precision spectroscopy. Although multi-scan averaging is the gold standard, its dependence on stationary samples and replicate measurements excludes real-time applications, while repeated scans compound readout noise—a critical limitation for transient phenomena.

These constraints demand algorithmic solutions. We systematically categorize and evaluate computational CRA removal methods, progressing from heuristic to model-based approaches.

### Moving average filtering

For single-scan spectral data where replicate measurements are unavailable, cosmic ray artifacts (CRAs) must be corrected in real-time. The moving average filter (MAF) achieves this by statistically detecting and excluding CRA-corrupted points before performing localized smoothing. While computationally efficient, MAF’s uniform-weight averaging inherently blunts legitimate sharp spectral features adjacent to CRAs—a limitation intrinsic to linear filtering approaches.

The algorithm first computes a first-order difference $\nabla Y_{t}=Y_{t}-Y_{t-1}$ to amplify CRA-induced transient spikes while suppressing low-frequency baseline drift. A modified *Z* score (Equation 1) then isolates these anomalies^30^:

(Equation 1) $$\left\{ \begin{array}{c} z_{t}=\frac{0.6745\times \left(\nabla Y_{t}-M\right)}{\text{MAD}}\\ \begin{array}{cc} M=\text{median}\left\{\nabla Y_{t}\right\}, & \text{MAD}=\text{median}\left\{\left|\nabla Y_{t}-M\right|\right\} \end{array} \end{array}\right.$$

where $Y_{t}$ represents the spectral intensity at wavenumber point t(t=2,3,…,n), the 0.6745 factor calibrates the median absolute deviation (MAD) to match the standard deviation of Gaussian-distributed noise. A threshold τ (typically 3–5) is applied to flag and exclude CRA-corrupted points from smoothing. The final filtered spectrum ${\tilde{Y}}_{t}$ is reconstructed via selective windowed averaging (Equation 2):

(Equation 2) $$\begin{array}{cc} {\tilde{Y}}_{t}=\frac{1}{\omega }\sum_{k=-m}^{m}Y_{t+k}\times I\left(\left|z_{t+k}\right|<\tau \right)\text{,} & w=\sum_{k=-m}^{m}I\left(\left|z_{t+k}\right|<\tau \right) \end{array}$$

where the indicator function I(·) ensures only non-CRA points contribute, and the half-width m adjusts smoothing intensity. This approach achieves real-time cosmic ray correction with robust artifact removal and computational efficiency, but trades off spectral resolution (near affected regions) and sensitivity to window size tuning.

### Missing-point polynomial filters

While MAF provides a foundational approach to spike rejection through linear averaging, the Missing-Point Polynomial Filter (MPF) advances this paradigm by strategically excluding CRA-corrupted points during polynomial regression, thereby preserving spectral features adjacent to artifacts with higher fidelity^31^:

(Equation 3) $$\left\{ \begin{array}{l} \begin{array}{cc} {\hat{y}}_{s+k}=\sum_{i=-m}^{m}C_{2,k,i}y_{s+k-i}, & y_{i}=\sum_{j=0}^{n}\beta_{n,i}i^{j}+\varepsilon_{i} \end{array}\\ \begin{array}{ccc} k=-m,\ldots ,m, & \vec{y}=X\vec{\beta }+\vec{\varepsilon }, & \vec{b}={\left(X^{\prime }X\right)}^{-1}X^{\prime } \end{array} \end{array}\right.$$

Unlike conventional Savitzky-Golay smoothing, which applies symmetric convolution to all points, MPF explicitly omits the central outlier (e.g., $y_{s}$) by enforcing zero convolution weights ($C_{2,0,i}=0$). By fitting a quadratic polynomial (n=2) to the remaining points within [s−m,s+m], MPF computes a corrected value ${\hat{y}}_{s}$ purely from neighboring data, minimizing distortion while retaining computational efficiency. The regression coefficients $\vec{\beta }={\left(\beta_{0},\beta_{1},\beta_{2}\right)}^{T}$ are derived via least-squares minimization, where X is the Vandermonde matrix of terms $i^{j}$ , and $\vec{\varepsilon }$ represents residuals.

This approach is particularly suited for isolated cosmic ray spikes in uniformly sampled spectra (e.g., Raman or IR), eliminating artifacts without the spectral broadening associated with MAF.

### Multistage spike recognition

Multistage spike recognition (MSR) algorithm^32^ improves upon standard cosmic ray removal methods (e.g., MPF) by integrating adaptive dynamic thresholds, iterative statistical validation, and signal-shape constraints. This automated technique accurately discriminates and corrects cosmic ray artifacts in time-resolved Raman spectra without distorting authentic chemical variations.

The algorithm processes spectral data $S_{\left(i,j\right)}$ (where i = temporal sampling points, j = wavenumbers) by first computing forward differences to enhance transient signals:

(Equation 4) $$\left\{ \begin{array}{l} D_{\left(k,m\right)}=S_{\left(k+1,m\right)}-S_{\left(k,m\right)}\\ \begin{array}{cc} k=1,\ldots ,i-1, & m=1,\ldots ,j \end{array} \end{array}\right.$$

Potential cosmic ray spikes are identified through iterative statistical filtering, where deviations in $D_{\left(k,m\right)}$ are compared against a dynamic threshold $t_{\left(1,j\right)}=4\sigma_{\left(1,j\right)}$. Here, $\sigma_{\left(1,j\right)}$ is the locally recalculated standard deviation after excluding outlier to avoid bias. False positives are minimized by requiring spikes to exhibit a sharp rise followed by an immediate return to baseline. For apodized spectra, a secondary threshold (empirically set to $0.5\sigma_{\left(1,j\right)}$) corrects spike shoulders while enforcing a maximum width constraint (≤30 pixels) to prevent overcorrection.

Validated across diverse experimental conditions, MSR demonstrates robust performance—particularly in datasets with 40–50+ sequential spectra—due to its adaptive thresholding, which reduces sensitivity to parameter selection and baseline drift.

### Polynomial filters

After MSR identifies cosmic ray spikes through adaptive thresholds and signal-shape constraints (e.g., sharp rise/fall within ≤30 pixels), polynomial filtering (PF)^33^ corrects the affected regions via localized polynomial interpolation. This automated method preserves underlying spectral features while minimizing artifacts by leveraging dataset-wide consistency. PF achieves this by:

(1) Global polynomial fitting – Fitting a third-order (or higher) polynomial across all spectra at each wavenumber to establish the expected intensity distribution.
(2) Outlier rejection – Flagging data points exceeding 3× the standard noise deviation as cosmic ray artifacts while retaining genuine Raman peaks.
(3) Parameter efficiency – Requiring only two adjustable parameters (polynomial order and threshold multiplier), enabling batch processing of homogeneous datasets.

For heterogeneous datasets, supplemental techniques (e.g., manual validation or median filtering) ensure accuracy without distorting true spectral features. PF’s robustness and minimal user intervention make it ideal for high-throughput Raman applications where manual inspection is impractical.

### Nearest neighbor comparison (NNC)

PF relies on global polynomial fits for CRA removal, whereas the nearest neighbor comparison (NNC)^34^^,^^35^ method enhances robustness by incorporating spectral covariance-based cross-validation, dynamically identifying anomalies through spatial comparison to maintain inter-spectral coherence in single-scan datasets. The algorithm quantifies spectral similarity using normalized covariance:

(Equation 5) $$C_{nm}=\frac{{\left(S_{n}\cdot S_{m}\right)}^{2}}{\left(S_{n}\cdot S_{n}\right)\left(S_{m}\cdot S_{m}\right)}$$

where, “·” denotes the dot product between spectra $S_{n}$ and $S_{m}$, with higher values indicating greater similarity. For each spectrum, NNC computes its closest match by maximizing $C_{nm}$, then estimates local noise characteristics via Savitzky-Golay (SG) smoothing:

(Equation 6) $$\sigma_{n}=\frac{1}{N}\sqrt{\sum_{k=1}^{M}{\left[S_{n}\left(k\right)-\bar{S_{n}\left(k\right)}\right]}^{2}}$$

Here, $\bar{S_{n}\left(k\right)}$ represents the SG-smoothed signal, and $\sigma_{n}$ is the noise standard deviation. CRAs are flagged where the raw-smoothed residual exceeds $5\sigma_{n}$ (primary threshold), while a secondary threshold at $2\sigma_{n}$ captures broader, low-intensity anomalies in neighboring pixels. Artifacts are replaced using values from the best-matched spectrum, ensuring minimal distortion of genuine features. Unlike multi-scan methods, NNC’s single-scan design eliminates cumulative read noise, optimizing efficiency without compromising signal integrity—critical for time-sensitive studies. The method’s dual-threshold approach balances sensitivity and specificity, while its automated, statistics-driven workflow minimizes false positives even in spectrally variable datasets when combined with preprocessing (e.g., baseline correction).

### Wavelet transform methods

Wavelet-transform methods complement spatial-correlation-based techniques like NNC by employing multi-resolution decomposition to isolate CRAs while preserving fine spectral features across frequency scales. These artifacts manifest as narrow, high-intensity distortions in Raman spectra, obscuring underlying chemical information.^36^ The hybrid approach integrates discrete wavelet transform (DWT) and k-means clustering (KMC): each spectrum undergoes n-level wavelet decomposition, splitting into approximation ($a_{j_{n},k}^{m}$) and detail coefficients ($d_{j,k}^{m}$), with decomposition depth n determined by $2^{n+3}\approx N$ (where N is spectral length). Unlike genuine Raman signals (characterized by Lorentzian/Gaussian/Voigt profiles), CR spikes generate random high-frequency wavelet coefficients. K-means clustering then minimizes intra-cluster variance^37^ to isolate outliers:

(Equation 7) $$J=\sum_{l=1}^{K}\sum_{i=1}^{n_{l}}{\left|x_{i}^{\left(l\right)}-c_{l}\right|}^{2}$$

where, $c_{l}$ represents cluster centroids and $n_{l}$ their coefficient counts. An adaptive threshold $r_{j}$—scaling linearly for broad Raman peaks or with a square root function for sharp features—ensures dynamic artifact detection. The algorithm incrementally increases cluster count $C_{\text{num}}$ until the largest cluster’s radius falls below $r_{j}$, classifying non-clustered coefficients as CR spikes; these are replaced with centroid values, while unclustered coefficients retain original values to preserve spectral diversity. Finally, inverse DWT reconstructs the corrected spectrum.

For enhanced detection of multi-pixel CR events, lag plots further discriminate artifacts: Raman peaks form lemniscate patterns along y=x, whereas CR-induced deviations ($D_{i}=y_{i+1}-y_{i}$) appear as statistical outliers. A DWT filter extracts residuals ($\delta_{i}=D_{i}-\hat{D}$) and the Allan deviation (ADEV) provides a noise-robust threshold^38^:

(Equation 8) $$ADEV=\sqrt{\frac{1}{2\left(N-2\right)}\sum_{i=1}^{N-1}{\left(D_{i+1}-D_{i}\right)}^{2}}$$

Spikes exceeding 3×ADEV (98% confidence) are interpolated, with adjacent points flagged to capture broader artifacts. This method excels for CR spikes narrower than Raman peaks but faces limitations in low-resolution datasets where their widths overlap. Automated parameter optimization ensures scalability for large spectroscopic datasets, offering a robust alternative to spatial-correction methods like NNC without requiring multi-scan averaging

### Kernel principal component analysis residual diagnosis

Wavelet transforms decompose signals in the frequency domain to isolate noise. In contrast, Kernel PCA Residual Diagnosis (KPCARD)^39^ adopts a machine learning framework, leveraging kernel-induced feature spaces to detect and correct nonlinear artifacts while preserving subtle spectral features—an essential capability for high-precision applications. The foundation of this approach lies in principle component analysis (PCA), which decomposes a spectral dataset $X\in R^{n\times p}$ (with n spectra and p channels) via singular value decomposition (SVD) of its covariance matrix $C=X^{T}X$ expressing the data as:

(Equation 9) $$X={\text{TP}}^{T}+E$$

where T contains scores, P denotes loadings (principal components), and E represents residuals. The principal components are derived from the eigenvalue problem:

(Equation 10) $${\text{Cv}}_{k}=\lambda_{k}v_{k}$$

where, $\lambda_{k}$ and $v_{k}$ correspond to explained variance and eigenvector directions, respectively.

However, while PCA excels at modeling smooth spectral variations, it falters with sharp CRAs due to its linearity constraint. To address this, KPCARD extends the framework to Kernel PCA (KPCA)^40^ by nonlinearly mapping the data into a high-dimensional space H via a Gaussian kernel:

(Equation 11) $$K_{ij}=\exp\left(-\frac{{\left\|x_{i}-x_{j}\right\|}^{2}}{2\sigma^{2}}\right)$$

where σ adjusts the sensitivity to spectral distances. KPCA then applies PCA in H, enabling the extraction of nonlinear principal components that better isolate CRAs from genuine spectral bands.

The residual diagnosis stage introduces a dual-threshold mechanism (global and local) to optimize artifact detection:

(Equation 12) $$\left\{ \begin{array}{l} t_{1-j}=\lambda_{1-j}\cdot cl_{upper\_j}\\ t_{2-ij}=\lambda_{2-ij}\cdot n_{ij\_orig} \end{array}\right.$$

where the global threshold $t_{1-j}$ leverages confidence intervals ($cl_{{\text{upper}}_{j}}$), while the local noise threshold $t_{2-ij}$ incorporates second-derivative estimates ($n_{ij\_orig}$), ensuring robustness against noise fluctuations. Residuals exceeding both thresholds are flagged as CRAs; corrupted regions are then reconstructed via:

(Equation 13) $$x_{ri}=ax_{nn}+b1+\sum_{k=1}^{r}c_{k}v_{k}$$

where $x_{nn}$ denotes a neighboring uncorrupted reference spectrum, a dynamically scales the intensity for spectral matching, b1 corrects baseline deviations, and $\sum c_{k}v_{k}$ reconstructs fine spectral features through a weighted ($c_{k}$) linear combination of retained principal components ($v_{k}$). By synergizing adaptive threshold detection with PCA-based reconstruction, this approach ensures robust artifact removal while faithfully preserving the intrinsic Raman spectral profiles.

### Cosmic ray removal: algorithm performance comparison

CRA correction methods span a spectrum balancing efficiency and precision. Rapid single-scan techniques (e.g., MAF) utilize statistical thresholding for real-time applications but may distort adjacent spectral features. Localized fitting approaches (e.g., missing-point polynomial filters, MPF) preserve fidelity by excluding corrupted points during interpolation, while multistage spike recognition (MSR) employs adaptive dynamic thresholds for robust iterative validation in time-resolved datasets.

For global optimization, PF fits homogeneous data efficiently but struggles with complex spectral variations. NNC leverages spectral covariance for artifact replacement, proving effective in dynamic systems. Wavelet-based methods excel in discriminatory power through multiscale decomposition and clustering, albeit requiring parameter tuning. At the highest precision tier, KPCARD enables nonlinear artifact detection at increased computational cost.

Method selection hinges on a triad of factors: artifact morphology (isolated spikes vs. distributed noise), dataset dynamics (static homogeneity vs. temporal variability), and accuracy-speed trade-offs—from rapid preprocessing (MAF/MPF) to critical applications requiring maximal feature preservation (wavelet/KPCARD).

## Baseline correction

After addressing high-frequency CRAs through thresholding or wavelet-based removal, spectroscopic preprocessing shifts to correcting baseline distortions—systematic low-frequency shifts caused by scattering, fluorescence, or detector drift. Unlike sparse CRA spikes, these broad interferences obscure true spectral features and skew quantitative analysis. Effective correction must disentangle analyte signals from background without distorting peak morphology, a task approached through physical/statistical models (e.g., polynomial fitting and asymmetric least squares), adaptive algorithms (e.g., penalized splines and iterative optimization), and machine learning (e.g., autoencoder-based detrending). The choice hinges on spectral complexity: simple baselines may succumb to linear models, while complex interferences demand data-driven adaptability. Below, we dissect these methods, balancing mathematical rigor with practical trade-offs.

### Wavelength artificial shift subtraction

Wavelength artificial shift subtraction (WASS) is a computational baseline suppression technique that isolates spectral signals by leveraging artificial wavelength shifts instead of empirical baseline fitting. The method decomposes an observed spectrum P(λ) into background B(λ) and signal S(λ):

(Equation 14) P(λ)=B(λ)+S(λ)

The key innovation lies in applying a small spectral displacement Δλ via a translation matrix T, generating a shifted spectrum $P^{\prime }=P\cdot T$. The difference between the original and shifted spectra is governed by a transfer matrix H:

(Equation 15) $$\begin{array}{cc} \Delta P=P-P^{\prime }=H\cdot P\text{,} & H=E-T \end{array}$$

where E is the identity matrix. The signal component S is then derived through least-squares minimization of the residual error:

(Equation 16) $$\hat{S}=\underset{S}{\mathrm{argmin}}{\left\|\Delta P-H\cdot S\right\|}^{2}$$

yielding the closed-form solution:

(Equation 17) $$S={\left(H^{T}H\right)}^{-1}\cdot H^{T}\Delta P$$

WASS is particularly effective in underwater LIBS, where it enhances faint emission lines obscured by broadband scattering.^41^ However, its performance hinges on careful calibration of Δλ—excessive shifts may amplify noise, while insufficient displacements fail to separate the baseline.

### Polynomial fitting

Unlike WASS, which relies on physical spectral shifts, polynomial fitting (PF) provides a model-free approach to baseline estimation. However, traditional single-polynomial fitting suffers from order sensitivity—high-order polynomials introduce oscillations, while low-order polynomials underfit complex baselines.^42^^,^^43^ Piecewise polynomial fitting (PPF) addresses this limitation by adaptively segmenting the spectrum and fitting independent polynomials to each region^44^^,^^45^:

(Equation 18) $$b=\left\{ \begin{array}{cc} \begin{array}{l} b_{1}+\alpha_{1}x+\cdots +\alpha_{p_{1}}x^{p_{1}}\\ b_{2}+\beta_{1}x+\cdots +\beta_{p_{2}}x^{p_{2}}\\ \vdots \\ b_{n}+\gamma_{1}x+\cdots +\beta_{p_{n}}x^{p_{n}} \end{array} & \begin{array}{l} x_{1}\leq x\leq {\text{seg}}_{1}\\ {\text{seg}}_{1}<x<{\text{seg}}_{2}\\ \vdots \\ {\text{seg}}_{n}<x<x_{n} \end{array} \end{array}\right.$$

where b is the fitted baseline, $x_{1},x_{n}$ define the spectral boundaries, ${\text{seg}}_{1},\ldots {\text{seg}}_{n}$ partition the spectrum, and $p_{1},\ldots ,p_{n}$ are individually optimized polynomial orders per segment.

Recent refinements, such as S-ModPoly, integrate sliding-window segmentation and iterative refinement, reducing processing times to <20 ms for Raman spectra while eliminating inter-segment discontinuities.^46^ PPF’s flexibility has proven especially powerful in chromatographic soil analysis, where coupling polynomial baselining with vector normalization achieves 97.4% accuracy in land-use classification.^47^

### B-spline fitting method

B-spline fitting (BSF) offers a flexible and robust approach to spectral baseline correction, combining low-degree polynomials with localized control to avoid overfitting while maintaining smoothness. Unlike traditional polynomial fitting, BSF decomposes the spectral domain into segments defined by a knot vector $T=\left\{t_{0},t_{1},\ldots ,t_{N+2k}\right\}$, ensuring each segment is influenced only by nearby control points.^48^ This localized adaptability makes BSF particularly effective in handling complex spectral variations, such as overlapping peaks and irregular baselines.

The BSF curve for an n- point dataset ${\left\{\left(x_{i},y_{i}\right)\right\}}_{i=1}^{n}$, is constructed using piecewise polynomial basis functions $B_{j,k}\left(x_{i}\right)$, where k denotes the polynomial degree (e.g., cubic splines with k=3). The fitted curve is expressed as ^48^^,^^49^^,^^50^:

(Equation 19) $$s_{i}=\sum_{j=0}^{N+k}c_{j}B_{j,k}\left(x_{i}\right)$$

where $c_{j}$ are control coefficients, and the basis functions are defined recursively. Starting from piecewise constant functions for k=0:

(Equation 20) $$B_{j,k=0}\left(x\right)=\left\{ \begin{array}{cc} 1 & t_{j}\leq x\leq t_{j+1}\\ 0 & \text{otherwise} \end{array}\right.$$

higher-order B-splines (k>0) are computed via:

(Equation 21) $$B_{j,k}\left(x\right)=\frac{x-t_{j}}{t_{j+k}-t_{j}}B_{j,k-1}\left(x\right)+\frac{t_{j+k+1}-x}{t_{j+k+1}-t_{j+1}}B_{j+1,k-1}\left(x\right)$$

The fitting process employs least-squares optimization to determine the optimal control points C (The control polygon formed by $C={\left[c_{0},c_{1},\ldots ,c_{N+k}\right]}^{T}$):

(Equation 22) $$\hat{C}={\left[P^{T}P\right]}^{-1}P^{T}Y$$

minimizing the error ${\left\|Y-\text{PC}\right\|}^{2}$, where P is the B-spline basis matrix and Y the observed spectral intensities.

By adjusting the knot density and polynomial degree, BSF balances smoothness and flexibility, outperforming conventional methods in scenarios like trace gas detection (demonstrating 3.7× higher sensitivity for NH_3_, O_3_, and CO_2_).^51^ Its local control prevents distortions from isolated noise spikes, while the recursive basis ensures computational efficiency—critical for processing large-scale spectral datasets.

### Two-side exponential fitting

The automatic two-side exponential (ATEB) method provides an effective solution for baseline correction through its bidirectional exponential smoothing approach. While B-spline fitting’s geometric flexibility makes it ideal for modeling smooth baselines, ATEB’s dynamic adaptation mechanism specializes in handling baseline drift while effectively eliminating edge artifacts, though it faces challenges with rapid signal fluctuations compared to localized methods like B-splines.

At the core of ATEB’s operation lies a weighted moving average process that self-adjusts based on spectral characteristics. The method calculates the baseline estimate $S_{t}$ at each point t through the recursive relation:

(Equation 23) $$\begin{array}{cc} S_{t}=\alpha x_{t}+\left(1-\alpha \right)S_{t-1}\text{,} & t>1 \end{array}$$

where $x_{t}$ represents the raw signal intensity and the smoothing factor α(0<α<1) controls the trade-off between noise suppression and baseline tracking responsiveness. Lower values of α provide stronger smoothing, while higher values allow quicker adaptation to baseline variations.

ATEB’s distinctive feature is its bidirectional processing, combining results from forward (from t=1 to n) and backward (from t=n to 1) passes to cancel out edge distortions that commonly plague one-sided smoothing methods. The algorithm iterates this process until the baseline stabilizes, typically when changes between iterations fall below a predefined threshold ε.

With linear time complexity O(n) per iteration, ATEB offers computational efficiency suitable for processing large multivariate datasets. Its completely automated operation eliminates the need for user-defined peak detection or prior knowledge of spectral features, making it particularly useful for high-throughput applications. It is effective in resolving complex baselines while preserving analytical signal integrity.^52^

However, the method’s reliance on averaging makes it less suited for signals with rapid fluctuations, where B-spline approaches with localized control demonstrate superior performance. This trade-off positions ATEB as an excellent choice for smooth or moderately varying baselines, while suggesting alternative methods might be preferable for more volatile spectral features.

### Morphological operations and mollification

Compared to ATEB’s statistical smoothing, which lacks spatial awareness, morphological operations—erosion and dilation—leverage a localized, topology-driven approach to signal processing. These operations excel in pharmaceutical spectral analysis, though their performance depends on carefully tuning the structural element (SE) width 2l+1.^53^ Rooted in set theory, mathematical morphology inherently captures geometric features, making it highly effective for tasks such as baseline correction.^53^^,^^54^^,^^55^^,^^56^^,^^57^^,^^58^ The four fundamental operators—erosion, dilation, opening, and closing—use a sliding window of 2l+1 points centered at $x_{i}$:

(Equation 24) $$\left\{ \begin{array}{l} \begin{array}{cc} \text{Erosion}\left(x_{i}\right)=\min\left(x_{i+j}\right), & j=-l,\cdots ,l \end{array}\\ \text{Dilation}\left(x_{i}\right)=\max\left(x_{i+j}\right)\\ \text{Opening}\left(x_{i}\right)=\text{Dilation}\left[\text{Erosion}\left(x_{i}\right)\right]\\ \text{Clo}\sin\;g\left(x_{i}\right)=\text{Erosion}\left[\text{Dilation}\left(x_{i}\right)\right] \end{array}\right.$$

Here, erosion suppresses narrow peaks while broadening valleys, whereas dilation enhances peak prominence while narrowing valleys. The opening operation (erosion followed by dilation) smooths signals by eliminating small fluctuations but may distort sharp peaks. Conversely, closing (dilation followed by erosion) fills gaps in the baseline but risks overestimation. To balance these effects, the Morphological Operation and Mollification (MOM) method computes their average:

(Equation 25) $$AVG\left(x_{i}\right)=\frac{\text{Opening}\left(x_{i}\right)+\text{Clo}\sin\;g\left(x_{i}\right)}{2}$$

To ensure smoothness, the estimated baseline $b_{i}\left(x\right)$ is convolved with a mollifier kernel M(r), an exponentially decaying function with compact support on (−1,1):

(Equation 26) $$M\left(r\right)=\left\{ \begin{array}{cc} \exp\left(\frac{-1}{1-r^{2}}\right), & \left|r\right|<1\\ 0, & \text{otherwise} \end{array}\right.$$

This normalized convolution refines the baseline while suppressing artificial oscillations. The smoothed baseline is expressed as follows:

(Equation 27) $${\text{smoothed}}_{b_{i}}\left(x\right)=\frac{\sum_{k=1}^{n}b_{i}\left(k\right)\cdot M\left(\frac{x-k}{2l+1}\right)}{\sum_{k=1}^{n}M\left(\frac{x-k}{2l+1}\right)}$$

The process terminates when either the maximum iterations are reached or the relative change rate (RCR) falls below a threshold (typically 10^−3^ to 10^−6^).

MOM has demonstrated strong performance in pharmaceutical applications, particularly in enhancing PCA-based classification^58^^,^^59^ of active compounds in tablets—provided the SE width matches the spectral feature sizes.

### SVD method

SVD provides an effective low-rank approximation framework for automated baseline correction in spectroscopic analysis. This approach fundamentally differs from morphology-based methods by employing orthogonal matrix decomposition to separate low-rank baseline variations from high-rank signal components. Given a spectral data matrix $Y\in R^{m\times n}$ (where m represents wavelength points and n denotes samples), SVD factorizes the matrix as:

(Equation 28) $$Y=U\sum V^{T}=\sum_{j=1}^{r}\sigma_{j}u_{j}v_{j}^{T}$$

where, U (left singular vectors) and V (right singular vectors) are orthogonal matrices, and ∑ contains the singular values $\sigma_{j}$ in descending order. The algorithm’s effectiveness stems from its ability to capture baseline artifacts in the first k singular vectors (associated with the largest $\sigma_{j}$). The reconstructed signal ${\hat{Y}}_{j}\left(\nu \right)$ aggregates high-rank residual components (from j=k+1 to r) as a weighted sum:

(Equation 29) $${\hat{Y}}_{j}\left(\nu \right)=\sum_{j=k+1}^{r}\sigma_{j}V_{ij}S_{j}^{\prime }\left(\nu \right)$$

where $\sigma_{j}$ scales significance, $V_{ij}$ are sample weights (from V), and $S_{j}^{\prime }\left(\nu \right)$ are baseline-corrected spectral basis functions (derived from truncated left singular vectors). Truncation at k isolates the true signal by excluding dominant baseline contributions.^59^

The decomposition explicitly separates the measured signal into baseline and corrected spectrum through:

(Equation 30) $$S_{j}\left(\nu \right)=S_{j}^{\prime }\left(\nu \right)+P_{j}\left(\nu \right)$$

where $P_{j}\left(\nu \right)$ represents the non-Raman background. Optimal k selection can be achieved via variance analysis or advanced methods like fuzzy generalized SVD (FGSVD),^60^ which achieves 100% classification accuracy in NIR spectroscopy. Compared to alternatives, SVD demonstrates superior performance in feature preservation—e.g., improving $R^{2}$ from 0.55 to 0.88 in DPPH/ABTS assays—while remaining computationally efficient for large datasets (e.g., GC-TOF-MS).^61^

### Wavelet transforms method

The wavelet transform (WT)^62^^,^^63^^,^^64^ overcomes the global spectral mixing of SVD by providing localized multiscale decomposition. In this framework, a signal x(t) is partitioned into low-frequency approximations ($A_{J}\left(t\right)$, representing baseline trends) and high-frequency details ($D_{J}\left(t\right)$, encoding peaks and noise) through the DWT. Mathematically, this is expressed as:

(Equation 31) $$x\left(t\right)=\sum_{j,k}a_{j,k}\phi_{j,k}\left(t\right)+\sum_{j,k}d_{j,k}\psi_{j,k}\left(t\right)=A_{J}\left(t\right)+D_{J}\left(t\right)$$

where the wavelet function $\psi_{j,k}\left(t\right)=2^{j/2}\psi \left(2^{j}t-k\right)$ extracts high-frequency components while the scaling function $\phi_{j,k}\left(t\right)=2^{j/2}\phi \left(2^{j}t-k\right)$ captures low-frequency dynamics. The coefficients $a_{j,k}$ and $d_{j,k}$ are computed via iterated filtering:

(Equation 32) $$\begin{array}{cc} a_{j,k}=\frac{1}{\sqrt{2}}\sum_{n}h_{n}c_{j+1,n+2k}\text{,} & d_{j,k}=\frac{1}{\sqrt{2}}\sum_{n}g_{n}c_{j+1,n+2k} \end{array}$$

with h (low-pass) and g (high-pass) constituting a two-channel filter bank designed for exact reconstruction, where $g\left[k\right]={\left(-1\right)}^{k}h\left[1-k\right]$ for orthogonal wavelets. Implemented via the Mallat algorithm,^65^ the DWT achieves exact reconstruction by recursively decomposing and reconstructing the signal through down-sampling and up-sampling operations. Crucially, this method requires no prior assumptions about baseline or peak morphology,^66^ enabling robust separation even for complex spectra. For example, in terahertz spectroscopy, the db4 wavelet reduced baseline RMSE to 0.57%—21-fold lower than polynomial fitting’s 12.38%.^67^ While minor artifacts (e.g., negative lobes^68^) can arise from wavelet oscillations, these are readily suppressed via thresholding, preserving the DWT’s dominance in noise-resistant spectral analysis.

### Least squares methods

Unlike wavelet-based methods that implicitly address baseline distortion, the weighted penalized least squares (wPLS) framework explicitly models baseline correction through a regularized optimization approach.^69^^,^^70^^,^^71^^,^^72^^,^^73^ This method is particularly effective for Raman and IR spectroscopy, where baseline drift often dominates noise. Given an observed spectrum $y={\left[y_{1},y_{2},\ldots ,y_{N}\right]}^{T}$ composed of the true signal s, baseline z, and random noise ϵ, the wPLS objective function balances fidelity term F amd roughness penalty R:

(Equation 33) $$F=\sum_{i=1}^{N}\omega_{i}{\left(y_{i}-z_{i}\right)}^{2}$$

(Equation 34) $$R=\sum_{i=1}^{N}{\left(\Delta^{2}z_{i}\right)}^{2}=z^{T}D_{2}^{T}D_{2}z$$

where $\omega_{i}$ are adaptive weights that downweight spectral regions with peaks or noise, ensuring baseline fidelity while mitigating undue influence from signal features, $D_{2}$ is a second-order difference matrix (a Toeplitz matrix with [1,2,1] diagonals) that enforces smoothness by penalizing sharp curvature in the baseline estimate z. The combined objective function is formulated as:

(Equation 35) $$Q=F+\lambda R={\left(y-z\right)}^{T}W\left(y-z\right)+\lambda z^{T}D_{2}^{T}D_{2}z$$

where λ is the regularization parameter, chosen via L-curve analysis^74^^,^^75^^,^^76^ or cross-validation to trade off baseline smoothness (R) against fitting accuracy (F). The closed-form solution is derived via normal equations:

(Equation 36) $$\hat{z}={\left(W+\lambda D_{2}^{T}D_{2}\right)}^{-1}Wy$$

Iterative reweighting refines W until convergence, typically controlled by a threshold $\epsilon \in \left[10^{-6},10^{-2}\right]$ on relative weight changes $\left|W_{t+1}-W_{t}\right|/\left|W_{t}\right|$.

One foundational advancement in this field is the Asymmetric Least Squares (AsLS) method,^77^ which employs asymmetric weighting to differentiate between peaks (signal) and baseline deviations. The weights $\omega_{i}$ are dynamically assigned as follows:

(Equation 37) $$\omega_{i}^{\text{AsLS}}=\left\{ \begin{array}{cc} p, & \begin{array}{cc} \text{if}\;y_{i}\geq z_{i} & \left(\text{Peaks},\text{heavily}\;\text{penalized}\right) \end{array}\\ 1-p, & \text{if}\;y_{i}<z_{i}\left(\text{baseline},\text{adaptively}\;\text{weighted}\right) \end{array}\right.$$

where p≫1−p. This asymmetry suppresses baseline drift while preserving high-frequency peaks. However, its reliance solely on second-order differences ($D_{2}$) limits flexibility in handling mixed-frequency artifacts (e.g., abrupt jumps or linear ramps).

The Improved AsLS (IAsLS)^78^^,^^79^ addresses this by combining first-order ($D_{1}$) and second-order ($D_{2}$) penalties:

(Equation 38) $$Q_{\text{IAsLS}}=\underset{\begin{array}{ccc} \text{weighted} & \text{fit} & \text{error} \end{array}}{\underset{︸}{\sum_{i=1}^{N}w_{i}{\left(y_{i}-z_{i}\right)}^{2}}}+\underset{\begin{array}{cc} \text{gradient} & \text{stability} \end{array}}{\underset{︸}{\lambda_{1}\sum_{i=1}^{N}{\left[\Delta \left(y_{i}-z_{i}\right)\right]}^{2}}}+\underset{\begin{array}{cc} \text{curvature} & \text{smoothness} \end{array}}{\underset{︸}{\lambda_{2}\sum_{i=1}^{N}{\left(\Delta^{2}z_{i}\right)}^{2}}}$$

where $D_{1}$ penalizes residual gradients to mitigate abrupt transitions (e.g., step artifacts), $D_{2}$ controls curvature to smooth slow drifts, $\lambda_{1}$ suppresses sharp jumps but may oversmooth weak peaks, and $\lambda_{2}$ flattens global curvature but risks attenuating genuine low-frequency signals. Optimal balance is empirically determined via cross-validation, prioritizing $\lambda_{1}$ for sharp artifacts (e.g., Raman baselines) and $\lambda_{2}$ for undulating offsets (e.g., NMR drift). IAsLS achieves local adaptability (peak preservation) and global smoothness (artifact rejection), making it robust across techniques with diverse distortion profiles.

To achieve superior peak symmetry in spectral baseline correction, the multiple constrained asymmetric least squares (mcaLS)^80^ method employs a tripartite objective function that simultaneously minimizes fitting residuals, penalizes excessive baseline curvature, and enforces symmetry in peak boundaries. The mathematical formulation is given by:

(Equation 39) $$Q_{\text{mcaLS}}=\sum_{i=1}^{N}\omega_{i}{\left(y_{i}-z_{i}\right)}^{2}+\lambda_{1}\sum_{i=2}^{N-1}{\left(\Delta^{2}z_{i}\right)}^{2}+\lambda_{2}\sum_{j=1}^{m}\sum_{k=1}^{n}{\left[\left(y_{Ljk}-z_{Ljk}\right)-\left(y_{Rjk}-z_{Rjk}\right)\right]}^{2}$$

Here, the first term represents a weighted sum of squared residuals, where $\omega_{i}$ assigns importance to each data point. The second term, regulated by the smoothing parameter $\lambda_{1}$, suppresses abrupt changes in the second derivative of the baseline ($\Delta^{2}z_{i}$), ensuring smooth approximation. The third term, controlled by $\lambda_{2}$, minimizes asymmetry in peak boundaries by equalizing the left ($y_{Ljk},z_{Ljk}$) and right ($y_{Rjk},z_{Rjk}$) residuals. When applied to maize near-infrared (NIR) spectra, mcaLS demonstrated superior performance, achieving RMSEs of 0.13 and 0.09 for quadratic polynomial and exponential baselines, respectively—significantly outperforming AsLS and ARPLS.^80^

For adaptive baseline fitting, the Adaptive Iteratively Reweighted Penalized Least Squares (airPLS)^69^^,^^72^ method employs a dynamic reweighting scheme that excludes peak regions while optimizing smoothness. Its objective function balances residual minimization against baseline roughness:

(Equation 40) $$Q^{t}=\sum_{i=1}^{m}w_{i}^{t}{\left|y_{i}-z_{i}^{t}\right|}^{2}+\lambda \sum_{j=2}^{m}{\left|z_{j}^{t}-z_{j-1}^{t}\right|}^{2}$$

Here, λ governs the smoothness penalty to prevent overfitting, while the adaptive weights ($w_{i}^{t}$) distinguish baseline and peak regions. At each iteration t, airPLS and its improved variant (IairPLS) update weights as follows:

(Equation 41) $${w_{i}^{t}|}_{\text{airPLS}}=\left\{ \begin{array}{cc} 0 & y_{i}\geq z_{i}^{t-1}\\ e^{\frac{t\left(y_{i}-z_{i}^{t-1}\right)}{\left|d^{t}\right|}} & y_{i}<z_{i}^{t-1} \end{array}\right.$$

(Equation 42) $${w_{i}^{t}|}_{\text{IairPLS}}=\left\{ \begin{array}{cc} 0 & y_{i}\geq z_{i}^{t-1}\\ \frac{te^{\frac{\left(y_{i}-z_{i}^{t-1}\right)}{\left|d^{t}\right|}}}{1+e^{\frac{\left(y_{i}-z_{i}^{t-1}\right)}{\left|d^{t}\right|}}} & y_{i}<z_{i}^{t-1} \end{array}\right.$$

The dynamic weighting mechanism operates in three critical steps: (1) peak exclusion (zero weight for $y_{i}\geq z_{i}^{t-1}$), (2) baseline refinement, where weights for points below the baseline ($y_{i}<z_{i}^{t-1}$)decay exponentially (airPLS) or follow a moderated sigmoid (IairPLS), scaled by the residual magnitude ($d^{t}$), and (3) termination when residuals fall below $\left|d^{t}\right|<0.001\left|y\right|$ or after maximal iterations.

IairPLS improves upon airPLS by smoothing transitions at peak boundaries, reducing artifacts in complex datasets. In X-ray fluorescence spectra of soil samples, IairPLS achieved an RMSE of 0.0187, outperforming not only mcaLS (0.3576), AsLS (0.6566), but also the original airPLS (0.0531).^69^ This highlights its robustness for applications demanding high-fidelity baseline correction.

The airPLS method, while effective for standard datasets, often underestimates baselines in non-peak regions and artificially inflates peak amplitudes when spectra are contaminated with noise. To address this limitation, the asymmetrically reweighted penalized least squares (arPLS)^81^ method was developed, employing a semi-balanced weighting strategy that accounts for noise distribution symmetry around peak-free baselines. Unlike airPLS, which aggressively suppresses all deviations, arPLS assigns intermediate weights to noise fluctuations near the baseline while zero-weighting signals that significantly exceed it (likely peaks). The weighting function is defined as:

(Equation 43) $$w_{i}^{t}=\left\{ \begin{array}{cc} \frac{1}{1+\exp\left\{2\left[\left(y_{i}-z_{i}\right)-\left(2\sigma_{d^{-}}-m_{d^{-}}\right)/\sigma_{d^{-}}\right]\right\}} & y_{i}\geq z_{i}^{t-1}\\ 1 & y_{i}<z_{i}^{t-1} \end{array}\right.$$

where $d^{-}$ denotes the negative residuals ($y_{i}-z_{i}^{t-1}$), and $\sigma_{d^{-}}$ and $m_{d^{-}}$ represent their standard deviation and mean, respectively. The logistic function $\log\text{istic}\left(x\right)=1/\left(1+e^{2x}\right)$ ensures gradual weight transitions, reducing abrupt artifacts. However, arPLS remains prone to overfitting faint noise in Raman spectra, occasionally misclassifying low-intensity peaks as baseline variations. To mitigate this, the inverse square root unit (ISRU) function^78^ was introduced as a replacement for the logistic component. ISRU’s smoother gradient better discriminates subtle features:

(Equation 44) $$w_{i}^{t}=\text{ISRU}\left(d_{i}\right)=\frac{1}{2}\left\{1-\frac{e^{t}\left(d_{i}-2\sigma_{d^{-}}\right)/\sigma_{d^{-}}}{\sqrt{1+{\left[e^{t}\left(d_{i}-2\sigma_{d^{-}}\right)/\sigma_{d^{-}}\right]}^{2}}}\right\}$$

Experimental validation on simulated Raman spectra (SNR = 30) confirmed ISRU’s superiority, achieving an RMSE of 7.71—significantly lower than arPLS (9.06), airPLS (10.25), and AsLS (9.49).^78^

For even greater precision, derivative-aware methods such as Adaptive Gaussian Derivative PLS (agdPLS)^82^ integrate spectral and baseline curvature penalties to distinguish peaks from noise. When applied to methane and ethane gas spectra, agdPLS increased $R^{2}$ values from 0.88 to >0.99 (methane) and 0.33 to >0.99 (ethane), showcasing its efficacy for gas-phase analysis. Further refinements emerged with the Spectral Estimation-based Asymmetrically Reweighted Least Squares (SEALS),^83^ which incorporates additive noise uniformity and signal energy distributions relative to the baseline. By dynamically adapting to local SNR conditions, SEALS achieves higher accuracy in low-SNR environments, making it indispensable for complex datasets.

Traditional baseline correction methods often suffer from empirical weight dependencies that render them sensitive to experimental variations and prone to inconsistent performance. To overcome these limitations, the Sparse Bayesian Learning (SBL) framework, also known as Bayesian reweighted penalized least squares (BrPLS), unifies spectral fitting and baseline correction through a probabilistic approach. Unlike conventional methods that approximate the $l_{1}$ norm through regularization, SBL directly addresses the fundamental $l_{0}$-norm minimization problem, achieving superior accuracy without requiring heuristic parameter tuning.^84^ The mathematical core of SBL employs Bayesian inference to derive adaptive weights^85^:

(Equation 45) $$w_{i}=\frac{1}{1+\frac{\beta }{1-\beta }\sqrt{\frac{\pi }{2}}F\left(x_{i}-z_{i},\sigma ,\mu \right)}$$

with the auxiliary function:

(Equation 46) $$F\left(d,\sigma ,\mu \right)=\frac{\sigma }{\mu }\left[1+\mathrm{erf}\left(\frac{d}{\sqrt{2}\sigma }-\frac{\sigma }{\sqrt{2}\mu }\right)\right]\exp\left[{\left(\frac{d}{\sqrt{2}\sigma }-\frac{\sigma }{\sqrt{2}\mu }\right)}^{2}\right]$$

In this formulation, the weight function $w_{i}$ provides a probabilistic measure of whether spectral data point $x_{i}$ belongs to the baseline $z_{i}$ or contains signal components. The parameter β(0<β<1) represents the prior probability of signal presence, while 1−β denotes baseline dominance likelihood. The error-function-based adaptation mechanism in F(d,σ,μ) enables automatic adjustment to local noise characteristics (σ being residual standard deviation) and signal amplitudes μ, preventing overfitting common in deterministic approaches.

Experimental validation demonstrates SBL/BrPLS’s exceptional performance, delivering $R^{2}$ values of 0.9990 for sinusoidal baselines compared to 0.9985 (arPLS), 0.8452 (airPLS), and 0.6222 (AsSL).^85^ This significant improvement stems from the framework’s ability to rigorously distinguish between noise and signal through statistical inference rather than amplitude thresholds, making it particularly robust against noise-dependent biases in spectral analysis.

### Sparse representation

While traditional baseline correction approaches like weighted least-squares methods (LSMs) achieve smoothing through curvature penalties, sparse representation techniques offer a fundamentally different paradigm—enforcing structural parsimony through adaptive dictionary learning, albeit with greater computational demands. This sparse representation framework has emerged as a particularly powerful approach for baseline correction, capitalizing on the inherent sparsity of analytical signals when projected onto properly designed dictionaries. The observed signal y is decomposed into three components:

(Equation 47) y=z+s+n

where z represents the low-frequency baseline, s captures structured signals (e.g., spectral peaks), and n accounts for noise. This decomposition is formalized in the simultaneous spectrum fitting and baseline correction via sparse representation (SSFBCSP)^86^ method as an optimization problem:

(Equation 48) $$\begin{array}{cc} \arg\min_{z,\alpha }{\left\|y-\Phi \alpha -z\right\|}_{2}^{2}+\lambda_{1}{\left\|Dz\right\|}_{2}^{2}+\lambda_{2}{\left\|\alpha \right\|}_{1}\text{,} & \alpha \geq 0 \end{array}$$

Here, s=Φα, with Φ being a dictionary (e.g., polynomial or wavelet basis), and α the sparse coefficient vector encoding the signal’s structure. The regularization terms $\lambda_{1}$ (smoothness control via differential operator D) and $\lambda_{2}$ (sparsity enforcement) balance fidelity and simplicity. While SSFBCSP excels in spectroscopy and biomedical applications due to its precision and noise robustness, its computational complexity remains a limitation for high-dimensional data.^86^

For instance, SSFBCSP achieves a prediction RMSE of 7.83 × 10^−4^ for Gaussian noise with sinusoidal baselines,^42^ outperforming alternatives like asLS (0.0062), airPLS (0.0052), IIPFAT (0.0104), CC (0.0094),^87^ and ATEB (0.0056).^52^ On the corn moisture dataset, it attains an $R^{2}$ of 0.9726, surpassing MSC (0.8881) and SNV (0.8082).^86^

To mitigate computational costs, downsampling^88^ techniques reduce data dimensionality without sacrificing accuracy. Leveraging Bayesian frameworks and independent vector decomposition, these methods decouple signal components adaptively and refine solutions via grid optimization, achieving performance comparable to sparse methods with lower overhead.^88^

Further advances include the fast burst-sparsity learning (FBSL-BC)^89^ method, which combines down-sampling for speed with burst-sparse modeling for accuracy. FBSL-BC employs a redundant dictionary of Voigt-like functions to represent spectral peaks:

(Equation 49) $$\left\{ \begin{array}{l} s\left(i\right)=\frac{c}{{\left[1+\beta^{2}\psi^{2}\left(i\right)\right]}^{\frac{1}{\beta^{2}}}}\text{,}\\ \begin{array}{cc} \psi \left(i\right)=\frac{i-i_{0}}{\sqrt{2}\sigma }, & i=1,2,\cdots ,N \end{array} \end{array}\right.$$

where, $i_{0}$ is the peak position, σ controls width, and β adjusts line shape (β=1 for Lorentzian, β=0 for Gaussian). By constructing Φ with parameter variations ($\sigma =\alpha^{j}$, $\Delta u=\alpha^{-1}$, etc.), FBSL-BC achieves 3–5× faster processing than traditional methods while maintaining high accuracy (e.g., $R^{2}=0.9647$ on corn NIR data^89^).

The Adaptive Sparse Decomposition Denoising (ASDD)^90^ algorithm dynamically builds dictionaries tailored to noise characteristics, demonstrating efficacy in applications like Raman spectroscopy for pesticide detection. These innovations highlight the trade-offs between accuracy, interpretability, and computational efficiency in modern baseline correction pipelines.

### Convex optimization framework

While sparse representation methods rely on manually designed dictionaries—limiting adaptability and requiring expert intervention—the Convex Optimization Framework (COF)^91^^,^^92^^,^^93^ provides a systematic alternative by unifying sparsity promotion, structural smoothness, and data fidelity through automated convex regularization. Unlike polynomial fitting or dictionary-based approaches, COF minimizes manual parameter tuning while preserving critical spectral features (e.g., Raman/LIBS peaks). Its core objective function F(x) is^94^:

(Equation 50) $$\hat{x}=\arg\min_{x}\left\{\begin{array}{l} F\left(x\right)=\frac{1}{2}{\left\|H\left(y-x\right)\right\|}_{2}^{2}+\\ \lambda_{0}\sum_{n=0}^{N-1}\theta_{\varepsilon }\left(x_{n};r\right)+\sum_{i=1}^{M}\lambda_{i}\sum_{n=0}^{N_{i}-1}\phi \left({\left[D_{i}x\right]}_{n}\right) \end{array}\right\}$$

The first term (data fidelity) ensures alignment with observed spectra, where $H=BA^{-1}$ acts as a high-pass filter. A is a baseline kernel and B extracts high-frequency features. The inversion $A^{-1}$ recovers suppressed high-frequency components by the baseline model.

The asymmetric penalty (adaptive sparse regularization) $\theta_{\varepsilon }\left(x_{n};r\right)$ dynamically switches between $l_{1}$-norm (sparsity) and $l_{2}$-norm (smoothness) via threshold ε and asymmetry parameter r:

(Equation 51) $$\theta_{\varepsilon }\left(x_{n};r\right)=\left\{ \begin{array}{cc} \begin{array}{l} \frac{1+r}{4\varepsilon }x^{2}+\begin{array}{c} x\\ \frac{1-r}{2}x\\ -rx \end{array}+\varepsilon \frac{1+r}{4} \end{array} & \begin{array}{l} x>\varepsilon \\ \left|x\right|\leq \varepsilon \\ x<-\varepsilon \end{array} \end{array}\right.$$

The third term of Equation 50 (structural preservation) penalizes noise while retaining peak structures using derivative operators $D_{i}$ (e.g., 1st/2nd differences) and a differentiable penalty ϕ(t)=|t|/(1+|t|/2).

COF leverages majorization-minimization (MM) to iteratively convexify the problem. The surrogate function G(x,v) simplifies computation via an adaptive weighting matrix Γ(ν) (diagonal entries ${\left[\Gamma \left(\nu \right)\right]}_{n,n}$ scale penalties by signal magnitude $\left|\nu_{n}\right|$) and a quadratic majorizer $\theta_{\varepsilon }^{\text{majorizer}}$ for $\theta_{\varepsilon }$:

(Equation 52) $${\left[\Gamma \left(\nu \right)\right]}_{n,n}=\left\{ \begin{array}{cc} \frac{1+r}{4\left|\nu_{n}\right|} & \left|\nu_{n}\right|\geq \varepsilon \\ \varepsilon & \left|\nu_{n}\right|\leq \varepsilon \end{array}\right.$$

(Equation 53) $$\theta_{\varepsilon }^{\text{majorizer}}\left(x_{n};r\right)=\left\{ \begin{array}{cc} \frac{1+r}{4\left|\nu \right|}x^{2}+\frac{1-r}{2}x+\varepsilon \frac{\left(1+r\right)\left|\nu \right|}{4} & \left|\nu \right|>\varepsilon \\ \frac{1+r}{4\varepsilon }x^{2}+\frac{1-r}{2}x+\varepsilon \frac{\left(1+r\right)}{4} & \left|\nu \right|\leq \varepsilon \end{array}\right.$$

The final surrogate combines all terms into a convex problem per iteration:

(Equation 54) $$G\left(x,v\right)=\left\{\begin{array}{l} \frac{1}{2}{\left\|H\left(y-x\right)\right\|}_{2}^{2}+\\ \lambda_{0}x^{T}\left[\Gamma \left(\nu \right)\right]x+\lambda_{0}b^{T}x+\\ \frac{1}{2}\sum_{i=1}^{M}\frac{\lambda_{i}}{2}{\left(D_{i}x\right)}^{T}\left[\Lambda \left(D_{i}v\right)\left(D_{i}x\right)\right]+c\left(v\right) \end{array}\right\}$$

Here, b introduces asymmetry, Λ weights derivative penalties for smoothness, and c(ν) provides a scalar offset.

In LIBS steel analysis, COF improved $R^{2}$ for Mn/Ni concentration predictions from 0.943/0.958 to 0.982/0.971,^94^ demonstrating superior baseline removal and feature retention compared to traditional methods.

### Bezier curve fitting

While COF’s mathematical rigor provides a systematic framework, Bezier curves offer parametric simplicity through iterative baseline interpolation using control points. This contrast is further enhanced by Bayesian approaches, which automate parameter selection by modeling signal sparsity probabilistically. Mathematically, a Bezier curve is defined as a parametric curve whose shape is determined by weighted combinations of Bernstein basis polynomials. Specifically, the position of an n-th degree Bezier curve p(t) at parameter t is given by^87^^,^^95^:

(Equation 55) $$\left\{ \begin{array}{l} p\left(t\right)=\sum_{i=0}^{n}b_{i}B_{i}^{n}\left(t\right)\\ \begin{array}{cc} B_{i}^{n}\left(t\right)=\left(\begin{array}{l} n\\ i \end{array}\right){\left(1-t\right)}^{n-i}t^{i}, & t\in \left[0,1\right] \end{array} \end{array}\right.$$

where, $B_{i}^{n}\left(t\right)$ are the Bernstein polynomials, $\left(\begin{array}{l} n\\ i \end{array}\right)$ is the binomial coefficient, and $b_{i}$ denotes the i-th control point. The curve exhibits three critical properties for optimization-based fitting: it exactly interpolates the endpoints $b_{0}$ and $b_{n}$, maintains endpoint tangents aligned with the control polygon edges, and lies strictly within the convex hull of $\left\{b_{i}\right\}$, guaranteeing stability when used as constraints in convex programs.

These properties make Bezier curves naturally compatible with regularized least-squares and iterative corner-cutting algorithms,^87^ enabling adaptive baseline estimation with guaranteed convergence. For example, in terahertz spectra of ErFeO_3_ (0.93–5.11 mm thickness), this approach achieves 49–52.6% noise reduction while preserving convexity constraints. However, B-splines offer superior adaptability in optimization-driven tasks due to local control and piecewise polynomial continuity, enabling targeted knot placement that suppresses ∼71% of noise without global constraints.^96^ This positions Bézier curves as a globally convex but less flexible alternative for spectral preprocessing when strict convexity is prioritized over local adaptability.

### Genetic algorithm

Unlike Bezier curves, which demand manual control point placement and user intervention to iteratively adjust baseline shapes, genetic algorithms (GA) automate the optimization process entirely through evolutionary fitness criteria. This eliminates subjectivity by dynamically balancing peak preservation and artifact removal via a formalized objective function F. For spectral analysis, F quantifies the trade-off between Raman peak retention ($A_{p}$) and baseline distortion ($A_{z}$)^97^^,^^98^:

(Equation 56) $$\left\{ \begin{array}{l} F=\frac{m_{1}}{m_{2}}\\ \begin{array}{cc} m_{1}=\frac{\log\left(n_{p}\right)}{A_{p}}+\frac{A_{z}t}{\log\left(n_{z}\right)}, & m_{2}=\frac{A_{p}}{\left(A_{z}t+A_{p}\right)} \end{array}\\ t=\frac{\max\left[I_{z}-\min\left(I_{z}\right)\right]}{\text{mean}\left(I_{n}\right)} \end{array}\right.$$

Here, the term t penalizes overcorrection by normalizing intensity variations against noise, a feature absents in Bezier’s fixed parametric approach. The automation and adaptability of GA deliver comprehensive improvements across three critical dimensions: analytical performance, processing efficiency, and real-world robustness.

In complex spectral analysis, GA achieves superior precision with an RMSE = 0.0004—significantly outperforming conventional methods like AsLS (0.028) or airPLS (0.0007).^99^^,^^100^ GA’s adaptive framework enables seamless integration with advanced analytical workflows, demonstrating 16% lower PLS errors in *Gibberella fujikuroi* studies^101^ and enabling high-precision agricultural predictions (e.g., GA-CARS synergy yielding $R^{2}=0.927$ for apple Brix values).^102^ The method’s robustness is further evidenced in clinical applications—when coupled with Savitzky-Golay smoothing, GA attains 5.7 μM detection limits for methotrexate serum with 15.6% RSD, surpassing Bezier implementations by 30–110% in both sensitivity and accuracy.^103^ Together, these advantages position GA as the preferred choice for modern, high-throughput spectral analysis where Bezier’s manual paradigm falls short.

### Deep learning method

Traditional baseline correction methods like GA rely on computationally intensive heuristic searches, often requiring manual parameter tuning and iterative optimization. In contrast, deep learning (DL) automates this process through hierarchical feature extraction, delivering superior classification accuracy with minimal preprocessing. DL revolutionizes spectral processing by leveraging neural networks’ unparalleled capacity to model complex nonlinear relationships directly from raw spectroscopic data.

1D convolutional neural networks (1D-CNNs)^104^ exemplify this paradigm, replacing heuristic searches with learned spectral filters. Each convolutional layer applies localized kernels $w_{ik}^{l-1}$ to input spectra $s_{i}^{l-1}$, followed by rectified linear unit (ReLU) activation (defined as f(z)=max(0,z))^105^:

(Equation 57) $$y_{k}^{l}=\text{ReLU}\left(b_{k}^{l}+\sum_{i=1}^{N_{l-1}}w_{ik}^{l-1}*s_{i}^{l-1}\right)$$

by minimizing MSE between predictions $y^{L}$ and target spectra $t^{p}$, enabling joint baseline removal and feature extraction:

(Equation 58) $$E^{p}=\sum_{i=1}^{N_{L}}{\left(y_{i}^{L}-t_{i}^{p}\right)}^{2}$$

In MIR breast tissue classification, this achieved 95.09% accuracy by modeling nonlinear variations directly from raw spectra.^106^

For highly variable baselines (e.g., XRF of alloys), the enhanced GAN (EGAN) framework outperforms deterministic methods by jointly optimizing its generator G and discriminator D through adversarial training. The generator G processes raw spectra x and noise z∼N(0,1) via convolutional layers with skip connections, producing baseline-corrected outputs. Meanwhile, the discriminator D enforces distributional consistency by minimizing a least-squares adversarial loss (LSGAN):

(Equation 59) $$\min_{D}V\left(D\right)=\left\{\begin{array}{l} \frac{1}{2}E_{x∼P_{data}\left(x,x_{c}\right)}{\left[D\left(x,x_{c}\right)-1\right]}^{2}+\\ \frac{1}{2}E_{x∼P_{data}\left(x_{c}\right)}{\left[D\left(G\left(z,x\right),x\right)\right]}^{2} \end{array}\right\}$$

Here, D learns to push real corrected spectra $x_{c}$ toward 1 and generated spectra G(z,x) toward 0 through squared-error-driven training. The discriminator distinguishes real corrected spectra $x_{c}$ from generated ones G(z,x), while the generator G optimizes a composite objective^107^:

(Equation 60) $$\min_{G}V\left(G\right)=\left\{\begin{array}{c} \frac{1}{2}E_{x,z}\left[{\left(D\left(G\left(z,x\right)\right)-1\right)}^{2}\right]+\\ \lambda {\left\|G\left(z,x\right)-x_{c}\right\|}_{1}+\beta {\text{Loss}}_{\text{local}} \end{array}\right\}$$

This equation combines $l_{1}$-norm reconstruction ($\lambda {\left\|G\left(z,x\right)-x_{c}\right\|}_{1}$) for global fidelity and local peak optimization ($\beta {\text{Loss}}_{\text{local}}$) to critical spectral regions. $\beta {\text{Loss}}_{\text{local}}$ is a specialized local peak loss, scaled by a factor of β=100, that prioritizes accurate correction by minimizing squared errors within a local range (±n channels) of each peak position $P_{i}$ (total P peaks):

(Equation 61) $${\text{Loss}}_{\text{local}}=\sum_{i=1}^{P}\sum_{j=-n}^{n}{\left(x_{c}\left[P_{i}-j\right]-G\left(z,x\right)\left[P_{i}-j\right]\right)}^{2}$$

EGAN achieved $R^{2}>0.99$ on XRF data,^107^ with further improvements via transfer learning from alloys to soil samples.

For error-sensitive applications, variational autoencoders (VAEs) probabilistically encode input spectra into a latent space by defining a Gaussian posterior distribution:

(Equation 62) $$Z∼N\left(\mu_{z},\sigma_{z}^{2}\right)$$

where $\mu_{z}$ and $\sigma_{z}^{2}$ are the mean and variance learned by the encoder. To preserve differentiability during training, sampling employs the reparameterization trick:

(Equation 63) $$\begin{array}{cc} Z=\mu \left(X\right)+\sigma \left(X\right)\epsilon \text{,} & \epsilon ∼N\left(0,1\right) \end{array}$$

Here, stochasticity is decoupled into a fixed noise variable ϵ, enabling gradient propagation through μ(X) and σ(X). The decoder then reconstructs a denoised spectrum ${\hat{X}}^{\prime }$ by modeling its distribution conditioned on Z and decoder weights $\omega_{g}$:

(Equation 64) $$g\left({\hat{X}}^{\prime }|Z,\omega_{g}\right)∼N\left({\hat{u}}_{x^{\prime }},{\hat{\sigma }}_{x^{\prime }}^{2}\right)$$

Here, ${\hat{u}}_{x^{\prime }}$ and ${\hat{\sigma }}_{x^{\prime }}^{2}$ quantify both the reconstructed signal and its uncertainty, critical for error-aware applications. Training optimizes the VAE loss $L_{AVE}$, which combines reconstruction error with Kullback–Leibler (KL) divergence to regularize the latent space:

(Equation 65) $$L_{AVE}=-E\left[\log\;g\left({\hat{X}}^{\prime }|Z,\omega_{g}\right)\right]+KL\left[f\left(Z|X,\omega_{f}\right)\|p\left(Z\right)\right]$$

For diagonal-covariance Gaussian latent variables, the KL term simplifies to:

(Equation 66) $$KL\left[f\|p\right]=\frac{1}{2}\sum_{i=1}^{N}\left(\sigma_{i}^{2}+u_{i}^{2}-1-\log\;\sigma_{i}^{2}\right)$$

A TinyML-VAE variant demonstrated real-time performance (NRMSE 0.53–0.66) on embedded magnetometers,^108^ highlighting edge-compatibility. While traditional VAE-based approaches suffer from spectral over-smoothing and parameter sensitivity, modern DL methods leverage domain-specific architectures and multitask training to achieve unprecedented precision and robustness.

For spectroscopic applications, an enhanced U-Net model integrates Learned Feature Fusion (LFF) and Residual Dense Blocks (RDBs) in a three-stage training framework (unpaired pretraining, synthetic data augmentation, and contrastive fine-tuning), achieving near-perfect regression ($R^{2}\approx 0.99$) while preserving critical spectral features.^109^ In seismic data processing, TraceNet combines synthetic multistage drifts with real earthquake records for automated baseline correction (MSE <10^−4^), effectively recovering displacement signals without filtering artifacts.^110^ Similarly, hybrid ResNet-U-Net architectures for Raman spectroscopy^111^^,^^112^ handle high-order polynomial baselines (up to 7th degree) and complex noise, reducing errors by >85% (RMSE: 5.40 × 10^−4^) compared to asPLS while maintaining robustness on real-world samples.

These advancements—automated processing, quantitative precision, and feature preservation—demonstrate the superiority of data-driven DL over conventional techniques across analytical domains, laying the foundation for adaptive, high-fidelity baseline correction in complex scenarios.

### Baseline correction: precision-automation trade-offs in algorithms

Traditional baseline correction methods span a spectrum of accuracy, automation, and computational trade-offs: parametric fits (e.g., polynomials and B-splines) offer simplicity but require careful parameter tuning, while adaptive least-squares methods (AsLS and arPLS) improve robustness through iterative reweighting yet struggle with complex baselines. WTs excel in multiscale separation but may introduce artifacts, and morphological operations efficiently remove narrow distortions using geometric filters yet lack flexibility. Advanced approaches address these limitations at higher computational costs—sparse representation and convex optimization balance accuracy and efficiency but face scalability challenges, while evolutionary algorithms (e.g., genetic algorithms) dynamically optimize corrections for intricate spectra at significant runtime expense. DL models (CNNs and GANs) automate correction via data-driven feature learning, achieving state-of-the-art accuracy at the cost of large datasets and interpretability.

In practice, hybrid methods that integrate complementary algorithmic strengths (e.g., combining mathematical operators with iterative optimization or data-driven modules) are increasingly adopted to bridge these gaps, enhancing adaptability, precision, and scalability for real-world deployment.

## Scattering correction

While baseline correction addresses low-frequency distortions to reveal true spectral features, complete signal recovery requires further accounting for scattering effects—a wavelength-dependent phenomenon that introduces intensity variations and obscures chemical information. Scattering correlations elucidate these perturbations, particularly distinguishing between Rayleigh scattering (d≪λ) and Mie scattering (d≈λ), which dominate wavelength-dependent noise and systematic calibration errors. To mitigate these effects, field-standard techniques like multiplicative scatter correction (MSC) were developed as foundational solutions, later advancing to more sophisticated approaches.

### Multiplicative scattering correction

MSC is a chemometric technique that empirically mitigates additive (e.g., baseline shifts) and multiplicative (e.g., path length variations) scattering effects in spectral data.^113^ The method follows a three-step workflow.^114^

Step 1 is reference spectrum calculation, where a scatter-free reference spectrum ${\bar{x}}_{k}$ is computed as the mean intensity across all samples N at each wavelength k:

(Equation 67) $${\bar{x}}_{k}=\frac{1}{N}\sum_{i=1}^{N}x_{ik}$$

Step 2 is linear regression correction, where each sample spectrum $x_{ik}$ is modeled as a linear transform of the reference:

(Equation 68) $$x_{ik}=c_{i}+b_{i}{\bar{x}}_{k}+e_{ik}$$

where $c_{i}$ (intercept) corrects additive effects, $b_{i}$ (slope) compensates for multiplicative effects, and $e_{ik}$ denotes residuals. The coefficients ${\hat{b}}_{i}$ and ${\hat{c}}_{i}$ are estimated via ordinary least squares (OLS) by minimizing the residual sum of squares (RSS) with respect to $b_{i}$ and $c_{i}$:

(Equation 69) $$\left({\hat{b}}_{i},{\hat{c}}_{i}\right)=\underset{b_{i},c_{i}}{\mathrm{argmin}}\sum_{k=1}^{p}\left(x_{ik}-c_{i}-b_{i}{\bar{x}}_{k}\right)$$

This yields the closed-form solutions expressed as follows:

(Equation 70) $$\begin{array}{cc} {\hat{b}}_{i}=\frac{\sum_{k}\left(x_{ik}-{\bar{x}}_{i}\right)\left({\bar{x}}_{k}-\bar{x}\right)}{\sum_{k}\left({\bar{x}}_{k}-\bar{x}\right)}\text{,} & {\hat{c}}_{i}={\bar{x}}_{i}-{\hat{b}}_{i}\bar{x} \end{array}$$

where ${\hat{b}}_{i}$ is the ratio of covariance between $x_{ik}$ and ${\bar{x}}_{k}$ to the variance of ${\bar{x}}_{k}$, and ${\hat{c}}_{i}$ adjusts for the mean offset.

Step 3 is spectral alignment, where the corrected spectrum $x_{ik}^{*}$ is derived by normalizing the original signal:

(Equation 71) $$x_{ik}^{*}=\frac{x_{ik}-{\hat{c}}_{i}}{{\hat{b}}_{i}}$$

MSC demonstrates robust performance in standardized applications, such as FTIR-based crude oil API gravity prediction ($R^{2}=0.944$)^115^ and moisture analysis in freeze-dried mushrooms ($R^{2}=0.928$).^116^ However, its linearity assumption limits effectiveness for complex samples (e.g., biological tissues with nonlinear scattering or multiple interferents), prompting the adoption of advanced alternatives.

### Extended multiplicative signal correction

Building upon the framework of multiplicative scatter correction (MSC), extended multiplicative signal correction (EMSC) addresses limitations in conventional MSC—such as its reliance on linear assumptions—by introducing additional correction terms tailored to complex samples (e.g., biological tissues or moisture-rich environments).^117^^,^^118^ For multicomponent systems, EMSC models the corrected spectrum $x_{\text{multiple}}\left(\tilde{\nu }\right)$ using^119^:

(Equation 72) $$x_{\text{multiple}}\left(\tilde{\nu }\right)=b\cdot \bar{x}\left(\tilde{\nu }\right)+\sum_{j=1}^{N}h_{j}\cdot \Delta k_{j}\left(\tilde{\nu }\right)+e\left(\tilde{\nu }\right)$$

Here, $\bar{x}\left(\tilde{\nu }\right)$ is the mean reference spectrum, b scales multiplicative effects (e.g., path length variations), $\Delta k_{j}\left(\tilde{\nu }\right)$ represents interfering signals (e.g., water vapor bands) with coefficients $h_{j}$ optimized via least-squares regression, and $e\left(\tilde{\nu }\right)$ captures residuals. This selectively removes confounders while preserving analyte-specific features (e.g., disease biomarkers).

For nonlinear baseline distortions (e.g., fluorescence in Raman spectroscopy), EMSC incorporates a polynomial model:

(Equation 73) $$x_{\sin\text{gle}}\left(\tilde{\nu }\right)=a+b\cdot \bar{x}\left(\tilde{\nu }\right)+\sum_{i=1}^{n}d_{i}{\tilde{\nu }}^{i}+e\left(\tilde{\nu }\right)$$

where a accounts for baseline shifts, $d_{i}$ are polynomial coefficients, and the optimal order n is determined through cross-validation to avoid overfitting. For systematic noise (e.g., temperature-induced peak shifts in NIR), EMSC integrates PCA-based corrections:

(Equation 74) $$x_{\text{OSM}}\left(\tilde{\nu }\right)=\left\{\begin{array}{l} a+b\cdot \bar{x}\left(\tilde{\nu }\right)+d_{1}\tilde{\nu }+\\ d_{2}{\tilde{\nu }}^{2}+\sum_{k=1}^{A}g_{k}p_{k}\left(\tilde{\nu }\right)+e\left(\tilde{\nu }\right) \end{array}\right\}$$

Here, $p_{k}\left(\tilde{\nu }\right)$ are PCA loadings, and $g_{k}$ their weights, with principal components (PCs) typically retained to explain >95% cumulative variance. Residuals $e\left(\tilde{\nu }\right)$ are further decomposed to unmask subtle spectral features:

(Equation 75) $$e\left(\tilde{\nu }\right)=\sum_{j=N+1}^{J}b\cdot c_{j}\Delta k_{j}\left(\tilde{\nu }\right)$$

In practice, EMSC achieves high fidelity: it distinguishes Crohn’s disease via FTIR spectra with 97% specificity^120^ and reduces temperature artifacts in polymers from $R^{2}>95\%$ to <1%.^121^ Its modular design enables protocol customization but demands careful parameter optimization and computational resources.

### Detrending

While EMSC addresses multiplicative, additive, and structured noise through polynomial and PCA-based corrections, detrending (DT) offers a specialized solution for scenarios where baseline drift dominates spectral disturbances. By removing low-frequency variations via polynomial fitting, DT calculates the corrected spectrum z(λ) as:

(Equation 76) $$\begin{array}{cc} z\left(\lambda \right)=y\left(\lambda \right)-B\left(\lambda \right)\text{,} & B\left(\lambda \right)=\sum_{i=0}^{N}a_{i}\lambda^{i} \end{array}$$

Here, y(λ) is the raw spectrum, z(λ) the detrended output, and B(λ) a polynomial baseline fitted via least-squares regression. The order N controls flexibility—higher orders model complex drifts but risk overfitting.

In NIRS analysis of pearl millet genotypes, DT combined with SNV improved nutrient prediction ($R^{2}=0.969-0.993$) using PLS models,^122^ while for Traditional Chinese Medicine, DT achieved 97.4% species identification accuracy (KNN).^123^ DT’s focused approach—ideally paired with complementary techniques—makes it indispensable for baseline correction in spectral analysis.

### Standard normal variate

While DT eliminates low-frequency baseline drift through polynomial fitting, standard normal variate (SNV) specifically targets higher-frequency, sample-specific variations by centering each spectrum around zero and scaling it to unit variance. This makes SNV particularly effective for datasets where sample-to-sample variability—such as scattering effects, amplitude shifts, or localized baseline deviations—dominates spectral differences.^124^

The method operates independently on each spectrum, ensuring robustness under heterogeneous conditions (e.g., varying scattering or baseline levels). Mathematically, given a raw spectrum $x=\left[x_{1},x_{2},\ldots ,x_{N}\right]$, SNV transforms each intensity value $x_{i}$ at wavelength i as:

(Equation 77) $$\begin{array}{cc} z_{i}=\frac{x_{i}-\bar{x}}{\sigma }\text{,} & \sigma =\sqrt{\frac{1}{N-1}\sum_{i=1}^{N}{\left(x_{i}-\bar{x}\right)}^{2}} \end{array}$$

Here, $\bar{x}$ is the sample-wise mean (averaged across wavelengths), and σ is the standard deviation. Although SNV resembles *Z* score normalization (ZSN) in form, its per-spectrum application and spectral-specific design differentiate it from global normalization approaches, as it avoids assumptions of uniform statistics across samples.

SNV excels in applications requiring localized spectral correction, such as geographical origin tracing (99% accuracy in Panax ginseng authentication via LIBS-SVM^125^) and defect detection (enhancing NIR-HSI water-absorption peaks at 1450 nm for apple bruise analysis^126^). However, excessive scaling may suppress discriminative features, as seen when geometric methods outperformed SNV in hyperspectral edge-bruise detection.

### Scattering correction: a benchmark of methods

Scattering correction techniques—such as MSC, EMSC, detrending (DT), and SNV—address spectral artifacts through distinct yet complementary approaches. MSC efficiently corrects linear additive and multiplicative scattering effects but lacks robustness against complex interferents. EMSC, an advanced variant of MSC, incorporates polynomial baselines, interferent modeling, and PCA-based denoising, making it particularly effective in biological systems (e.g., serum or tissue spectra), though it requires careful parameter optimization. DT specifically targets baseline drift by fitting a low-order polynomial, offering a simple yet flexible solution for spectral detrending. SNV, on the other hand, normalizes each spectrum independently by scaling to zero mean and unit variance, which is highly effective for heterogeneous datasets (e.g., LIBS or NIR-HSI) but may overcorrect subtle spectral features.

The choice of method depends on the dominant spectral artifacts: MSC for basic scattering, EMSC for complex interferents or nonlinear effects, DT for baseline shifts, and SNV for sample-specific variations. For challenging datasets, hybrid approaches (e.g., EMSC + DT) often yield superior results by leveraging the strengths of multiple techniques. No single method universally dominates, emphasizing the need for method selection based on spectral characteristics and analytical objectives.

## Data normalization

While scattering correction methods address wavelength-dependent intensity distortions, spectral normalization techniques standardize sample-specific scaling effects, enabling robust comparisons across heterogeneous datasets.

In fields ranging from agricultural monitoring to pharmaceutical quality control, spectral variability arises from diverse sources—including measurement conditions, instrument response, and sample heterogeneity. These variations manifest as intensity shifts, baseline deviations, and variance disparities, which can critically degrade the performance of machine learning models during training and inference. Effective normalization addresses three core challenges: (1) preserving relative spectral features while standardizing numerical scales, (2) accelerating optimization convergence, and (3) enhancing model generalizability by reducing systematic biases.

The following sections systematically detail nine essential normalization methods, categorized by their mathematical principles and interdependent applications.

### Mean centering

Mean centering normalization (MCN)^127^ aligns spectral baselines by subtracting the arithmetic mean of the dataset from each data point. For a spectral vector $x=\left[x_{1},x_{2},\ldots ,x_{N}\right]$, the MCN-transformed value $z_{i}$ is computed as:

(Equation 78) $$\begin{array}{cc} z_{i}=x_{i}-\bar{x}\text{,} & \bar{x}=\frac{\sum_{i=1}^{N}x_{i}}{N} \end{array}$$

where $\bar{x}$ is the mean intensity across all wavelengths and N is the spectral data length.

By removing global intensity offsets, MCN mitigates spectrometer drift and baseline shifts while retaining relative spectral features—a property critical for PCA, where centering ensures principal components reflect true variance rather than artificial offsets.^128^^,^^129^ This is exemplified in agricultural NIR spectroscopy, where MCN preprocessing elevated prediction accuracy beyond 99.7% by accentuating wavelength-specific crop quality signatures,^130^ and in pharmaceutical quality control, where it resolved overlapping peaks in complex formulations (e.g., sodium hyaluronate mixtures) with correlation coefficients exceeding 0.999.^131^

### Orthogonal signal correction

Orthogonal signal correction (OSC)^132^^,^^133^^,^^134^^,^^135^^,^^136^ extends MCN’s variance-centric philosophy into multivariate space, addressing a critical limitation: while MCN removes global offsets, OSC systematically eliminates signal components orthogonal to target variables (e.g., analyte concentrations), leading to more robust modeling.

The Direct OSC (DOSC)^137^ algorithm accomplishes this through a three-step projection and decomposition process. First, the target response variable Y (e.g., concentration) is decomposed into its spectral-space projection $\hat{Y}$ and an orthogonal residual F:

(Equation 79) $$\hat{Y}=X{\left(X^{T}X\right)}^{-1}X^{T}Y$$

where $\hat{Y}$ represents the predictable variations in Y captured by X, while F contains noise unrelated to X. Crucially, the same decomposition is applied to the spectral data X itself, splitting it into a component aligned with $\hat{Y}$ and an orthogonal subspace Z:

(Equation 80) $$Z=\left(I-\hat{Y}{\hat{Y}}^{†}\right)X$$

Here, Z (satisfying $Z^{T}\hat{Y}=0$) isolates spectral variations uncorrelated with Y, and ${\hat{Y}}^{†}$ denotes the Moore-Penrose pseudoinverse, ensuring numerical stability in ill-conditioned cases.

To extract and remove structured noise, DOSC applies PCA/SVD to Z, deriving its dominant variations (U). These are then mapped back to the original data space through a correction weight matrix W, computed as:

(Equation 81) $$\begin{array}{cc} W=X^{†}U\text{,} & U_{\text{new}}=\text{XW} \end{array}$$

where $X^{†}$ again ensures robust inversion. The final projection loadings (P) for noise subtraction are given by:

(Equation 82) $$P=X^{T}U_{\text{new}}{\left(U_{\text{new}}^{T}U_{\text{new}}\right)}^{-1}$$

With these, the corrected spectral data ($X_{\text{DOSC}}$) is obtained by removing the noise subspace:

(Equation 83) $$X_{\text{DOSC}}=X-U_{\text{new}}P^{T}$$

New prediction samples ($X_{\text{pre}}$) are transformed analogously, retaining consistency between training and future data. The correction first projects $X_{\text{pre}}$ onto the orthogonal space to capture Y-irrelevant variations via:

(Equation 84) $$U_{\text{pre}}=X_{\text{pre}}W$$

Here, $U_{\text{pre}}$ captures the interference components in $X_{\text{pre}}$ aligned with the training set’s orthogonal subspace Z. Then removes them by:

(Equation 85) $$X_{\text{pre}}^{\text{DOSC}}=X_{\text{pre}}-U_{\text{pre}}P^{T}$$

where $X_{\text{pre}}^{\text{DOSC}}$ is the corrected spectral data ready for prediction.

The correction method demonstrates broad spectroscopic utility, transforming predictive accuracy across applications: By projecting data into orthogonal space ($X_{\text{pre}}\to U_{\text{pre}}$) and removing interference ($X_{\text{pre}}^{\text{DOSC}}$), it elevates rice bran oil analysis ($R^{2}:0.50\to 0.987$) through humidity elimination,^138^ enables single-component glucose prediction in bioreactors,^139^ and enhances soil monitoring ($R^{2}+0.259$, RMSE↓3.203 g/kg).^140^^,^^141^ While outperforming univariate approaches, its computational overhead necessitates tradeoff evaluation in resource-constrained settings.

### Min-max normalization

While methods like OSC selectively remove structured noise through orthogonal projections, min-max normalization (MMN) operates as a domain-agnostic scaling tool, universally bounding data ranges without distinguishing signal from interference. MMN is a linear transformation method that preserves relative feature distributions by mapping data to [0,1]^127^^,^^142^:

(Equation 86) $$z_{i}=\frac{x_{i}-x_{\min}}{x_{\max}-x_{\min}}$$

Its simplicity belies broad applicability: Raman-PLS models achieve 97% honey origin classification accuracy (vs. 85% unscaled),^143^ random forests identify microplastics at 99% precision,^144^ Terahertz-PLS models predicted caffeine/quinic acid/niacin mixtures with 0.0866 RMSE reduction,^145^ and biomedical models (e.g., heart disease detection^146^ and stress level analysis^147^) benefit from standardized intensities. However, MMN’s dependency on extremal values ($x_{\min}$, $x_{\max}$) renders it susceptible to outlier distortion—a limitation absent in OSC’s covariance-aware decomposition.

### *Z* score normalization

ZSN^148^ addresses the outlier sensitivity of MMN by standardizing data based on distributional statistics rather than extreme values. It transforms features to have a mean of 0 and variance of 1. For a dataset $x=\left[x_{1},x_{2},\ldots ,x_{N}\right]$, ZSN generates the normalized vector z:

(Equation 87) $$\begin{array}{cc} \begin{array}{cc} z_{i}=\frac{x_{i}-\bar{x}}{\sigma }, & z=\frac{x-\bar{x}\cdot I}{\sigma }, \end{array} & \sigma =\sqrt{\frac{1}{N}\sum_{i=1}^{N}{\left(x_{i}-\bar{x}\right)}^{2}} \end{array}$$

where $I={\left[1,1,\ldots ,1\right]}^{T}$ is an N-dimensional unit vector, σ is the population standard deviation. ZSN projects data onto a hyperplane orthogonal to I and scales it to a hypersphere of radius $\sqrt{N}$, enforcing:

(Equation 88) $$\begin{array}{cc} \left\|z\right\|=\sqrt{N}\text{,} & \left\langle z,I\right\rangle =0 \end{array}$$

This ensures mean-centered, unit-variance distributions while mitigating biases from unknown minima/maxima or singularities (e.g., spectroscopic scatter effects).

ZSN’s robustness is demonstrated across diverse domains. In spectroscopy, it enables PLS/RF-based tea identification^149^ and 2D stellar classification via attention mechanisms,^150^ while simultaneously eliminating scatter artifacts caused by particle heterogeneity in diffuse reflectance studies. In medical imaging, ZSN achieves superior cross-scanner harmonization of radiomic features (*p* < 0.001),^151^ and its noise suppression capability helps resolve multicollinearity in high-dimensional data.^124^ Additionally, the method’s mathematical simplicity and compatibility with localized adaptations (e.g., LSNV^152^) contribute to its analytical precision.

### 3-Sigma normalization

While ZSN standardizes data to a zero-mean, unit-variance distribution, 3-Sigma Normalization (3-SN) implements a more robust heuristic by truncating values outside $\bar{x}\pm 3\sigma$ and linearly transforming the inlier range to [0,1] via:

(Equation 89) $$z_{i}=\frac{x_{i}-\left(\bar{x}-3\sigma \right)}{6\sigma }$$

Here, $\bar{x}$ and σ denote the dataset’s mean and standard deviation. The numerator centers each $x_{i}$ relative to the lower 3σ bound, while the denominator scales the result by the total inlier range (6σ).

This dual mechanism—statistical truncation followed by linear scaling—proves invaluable in clinical spectroscopy, where it achieves 97.8% accuracy in breast tumor classification^153^ while mitigating scanner-to-scanner variability.^127^ By suppressing technical noise (e.g., instrumental artifacts) and preserving biological signals, 3-SN offers a principled balance between outlier resilience and data harmonization, complementary to ZSN’s Gaussian-centric approach.

### $l_{1}$ normalization

While statistical scaling methods (e.g., *Z* Score, 3-Sigma) focus on distributional properties, vector space methods like $l_{1}$ Normalization operate on geometric constraints. By scaling components relative to the Manhattan norm (sum of absolute values), $l_{1}$ Normalization preserves sparsity patterns—a critical requirement in applications such as non-intrusive load monitoring (NILM). Its formulation is given by:

(Equation 90) $$\begin{array}{cc} z_{i}=\frac{x_{i}}{{\left\|x\right\|}_{1}}\text{,} & {\left\|x\right\|}_{1}=\sum_{j=1}^{N}\left|x_{j}\right| \end{array}$$

Here, $z_{i}$ is the normalized value of the i-th feature, $x_{i}$ is the raw value, and ${\left\|x\right\|}_{1}$ (the Manhattan or Taxicab norm) computes the sum of absolute values across all features.

This approach is particularly effective in NILM, where appliance signatures often exhibit sparse energy consumption patterns (e.g., inactive phases with zero current). By preserving these zero-value components through $l_{1}$ scaling, the method achieves 96.42–97.65% accuracy on benchmark datasets (PLAID/WHITED).^154^

### Vector normalization ($l_{2}$ normalization)

Normalization techniques like $l_{1}$ and $l_{2}$ address scale invariance but prioritize different properties: While $l_{1}$ normalization (sum of absolute values) enforces sparsity by favoring feature selection, $l_{2}$ normalization (Euclidean norm) preserves directional relationships by projecting vectors onto a unit hypersphere—this makes it ideal for comparing angular similarity (e.g., cosine distance) rather than magnitude. Mathematically, $l_{2}$ normalization scales a vector x by its Euclidean norm:

(Equation 91) $${\begin{array}{cc} z_{i}=\frac{x_{i}}{{\left\|x\right\|}_{2}}, & {\left\|x\right\|}_{2}=\left(\sum_{j=1}^{N}x_{j}^{2}\right) \end{array}}^{\frac{1}{2}}$$

Its discriminative power is evident in diverse fields: Combined with probabilistic PCA (PPCA), it achieves 100% accuracy in brain MR classification (5×5-fold cross-validation) by enhancing directional contrast,^155^ while in agricultural spectroscopy, it enables non-destructive kiwifruit sugar prediction ($R^{2}=0.805$) through the VN-CARS-PLSR model, reducing spectral data volume by 88.6% without sacrificing accuracy (RMSEP = 0.498).^156^

### Logarithmic transformation

While $l_{1}$/ $l_{2}$ normalization achieves scale invariance through linear operations, logarithmic transformation (LT) resolves nonlinear distortions by converting multiplicative relationships into additive space—particularly valuable for spectral data with exponential dynamics or heavy skewness. The transformation is defined as:

(Equation 92) $$z_{i}=\log_{b}\left(x_{i}+c\right)$$

where b denotes the logarithmic base (typically 10 or e) adjusts scale sensitivity (with base e natural for theory-driven models and base 10 preferred for human-readable scales), and c (>0) ensures stability at $x_{i}=0$. By compressing dynamic ranges and suppressing extreme values, LT stabilizes variance and promotes normality, with demonstrated success in pulp quality analysis (RMSEP = 128, NIR absorbance)^157^ and soil organic carbon prediction ($R^{2}=0.79$).^158^ Unlike $l_{2}$ normalization’s focus on directional consistency, LT prioritizes distributional symmetry, though its requirement of $x_{i}+c>0$ may necessitate alternatives (e.g., Box-Cox) for non-positive or power-law-optimized data.

### 1/n Power transformation

Compared to $l_{2}$ normalization (directional scaling) or log transforms (multiplicative symmetry), the 1/n power transformation (1/n-PT) provides parametric control over value compression, balancing flexibility against data constraints. The transformation is defined as:

(Equation 93) $$z_{i}=x_{i}^{\frac{1}{n}}$$

where $n\in Z^{+}$ determines compression strength: larger n (e.g., n=2 for square roots) yields milder adjustments. By nonlinearly suppressing large values, 1/n-PT mitigates outliers and normalizes right-skewed distributions, proving advantageous for regression (variance stabilization) and machine learning (feature distribution optimization). However, its limitations include a requirement for $x_{i}\geq 0$, necessitating data shifting (e.g., $x_{i}+c$) for negative values. Moreover, its compression efficacy depends on n, necessitating careful hyperparameter tuning.

In spectroscopic analysis, 1/n-PT improves Cu(II)-DOM binding quantification. Coupled with biological treatment, it achieves 89% TOC removal, 55% humic substance enrichment, and enhanced complex stability, collectively mitigating environmental hazards.^159^

### D**ata normalization: linear, geometric, and nonlinear approaches**

Normalization techniques optimize data for downstream analysis by addressing scale, distribution, and noise, with nine prominent methods falling into three functional categories.

For linear adjustments, mean centering (MCN) removes baseline offsets and is fundamental to PCA, though it ignores scale standardization. Building on this, MMN constrains values to [0,1] for intuitive scaling but suffers under outliers—a weakness addressed by *Z* Score (ZSN), which enforces μ=0 and σ=1 through statistical scaling. To further robustify ZSN, 3-Sigma (3-SN) applies probabilistic Winsorization, automatically clipping extreme deviations beyond ±3σ. OSC extends linear approaches by projection pursuit, stripping multivariate noise orthogonal to targets (e.g., correcting spectral interferents), though at higher computational cost.

When geometric properties dominate, $l_{1}$ normalization prioritizes sparsity via Manhattan distances (key for feature selection), while $l_{2}$ normalization preserves directional relationships through Euclidean norms, proving superior in tasks like spectral discrimination.

Nonlinear dynamics demand specialized transforms: Logarithmic (LT) compression handles exponential-scale data (e.g., pixel intensities or highly skewed features), whereas 1/n-PT stabilizes heteroscedastic variance in regression via parametric scaling—e.g., square roots (n=2) for Poisson-like distributions.

Method selection balances data traits and analytical goals: outlier resilience favors ZSN or 3-SN over MMN; high-dimensional noise necessitates OSC; nonlinear skewness calls for LT or 1/n-PT; and geometric needs dictate $l_{1}$ (sparsity) versus $l_{2}$ (directionality). Real-world pairings include MMN+LT for image preprocessing, $l_{2}$ +LT for NLP embeddings, and ZSN+3-SN for environmental sensor normalization, each combo addressing domain-specific challenges.

## Filtering and smoothing

Building upon normalized spectral data (scaled and distribution-optimized via *Z* score, OSC, etc.), filtering and smoothing methods form the subsequent critical layer of preprocessing. These techniques target structured noise—high-frequency fluctuations, baseline drift, or stochastic artifacts—while preserving spectral fingerprints (e.g., peak positions, relative intensities, and band shapes) essential for downstream tasks like classification or regression. Here, we systematically present seven core methods, ordered by computational complexity and methodological interdependencies. Each approach negotiates the trade-off between noise suppression and feature fidelity, with selection guidelines provided for diverse spectroscopic modalities (e.g., Raman, NIR, and MS).

### Gaussian filtering

Gaussian filtering (GF) applies weighted averaging via a Gaussian kernel G(x) to suppress high-frequency noise while preserving spectral signatures:

(Equation 94) $$G\left(x\right)=\frac{1}{\sqrt{2\pi \sigma^{2}}}e^{-\frac{x^{2}}{2\sigma^{2}}}$$

The kernel’s width (σ) directly controls the trade-off between noise attenuation and feature distortion—smaller σ retains sharp peaks but may leave residual noise, whereas larger σ risks oversmoothing.

In practice, GF operates as a convolution-based low-pass filter, with its cutoff frequency $f_{c}\approx 1/\left(2\pi \sigma \right)$ naturally excluding high-frequency artifacts (e.g., detector noise). For LIBS data, σ proved critical for resolving emission lines (e.g., Li I 670.8 nm)—a value too small fails to attenuate noise, while too large merges adjacent peaks. When combined with stacked correlation feature selection, this approach enabled 100% accuracy in Dendrobium species classification—outperforming SVM/RF/KNN by 10–20%.^160^ Further, GF’s integration with baseline removal and normalization ensured robust mineral classification (e.g., lithium ores in complex matrices^161^), underscoring its versatility in spectral preprocessing.

### Savitzky-Golay filtering

Savitzky-Golay filter (SGF) extends GF’s concept by fitting local degree-*N* polynomials within a sliding window of 2M+1 points. Its smoothed output at point n is the central value of the fitted polynomial p(k)^162^^,^^163^:

(Equation 95) $$y\left[n\right]=\sum_{m=-M}^{M}h\left[m\right]x\left[n-m\right]$$

where the finite impulse response (FIR) coefficients h[m] derive from least-squares regression on the Vandermonde matrix A of polynomial bases $\left(1,k,k^{2},\ldots ,k^{N}\right)$ , computed as the 0th row of ${\left(A^{T}A\right)}^{-1}A^{T}$. The filter preserves spectral moments up to order N, unlike GF’s fixed spectral attenuation. The trade-offs are controlled by window size (M) and polynomial degree (N). Larger M enhances noise suppression but blurs transient features (cf. GF’s σ parameter), while higher N retains sharp peaks but overfits noise (GF lacks this adaptability).

Applications leverage SGF’s balance between noise removal and feature fidelity. It achieves 2.35× SNR enhancement in methane detection (LITES),^164^ improves soil phosphorus prediction ($R^{2}=0.81$ with adaptive MRLFOSGF^165^) in Vis-NIR datasets,^166^ reduces CO spectral errors from 25.2% to 5.9% (Vis-NIR),^167^ and maintains 0.53 ppm accuracy in TDLAS gas monitoring,^168^ with its noise suppression and peak-preserving capabilities making it indispensable across spectroscopy and industrial sensing applications.

### Wiener filtering

While SGF excels in local smoothing and spectral peak preservation, Wiener filtering (WF) provides a statistically optimal framework by minimizing the total mean-square error (MSE) across all frequencies:

(Equation 96) $$\varepsilon^{2}=\sum_{\nu }E\left[{\left|F\left(\nu \right)-\hat{F}\left(\nu \right)\right|}^{2}\right]$$

where $\hat{F}\left(\nu \right)=D\left(\nu \right)W\left(\nu \right)$ defines the estimated signal. For a noisy observation D(ν)=F(ν)H(ν)+N(ν) (with system distortion H(ν) and noise N(ν)), WF’s closed-form solution adaptively suppresses degradation by incorporating both the system response and local SNR^169^:

(Equation 97) $$\left\{ \begin{array}{l} W\left(\nu \right)=\frac{H^{*}\left(\nu \right)}{{\left|H\left(\nu \right)\right|}^{2}+\frac{P_{nn}\left(\nu \right)}{P_{ss}\left(\nu \right)}}\\ {\hat{F}}_{\text{restored}}\left(\nu \right)=D\left(\nu \right)W\left(\nu \right) \end{array}\right.$$

where it explicitly leverages signal and noise power spectra ($P_{ss},P_{nn}$). Assuming F(ν) and N(ν) are uncorrelated (E[F(ν)N∗(ν)]=0), WF outperforms heuristic methods like SGF in noise-modeled scenarios.

In precision spectroscopy, WF reduced CH_4_ detection limits from 150 ppm to 100 ppm (vs. SGF’s 120 ppm),^170^ while in hyperspectral imaging, it achieved comparable SNR (17 dB) with superior robustness to non-uniform noise^171^; For miniature spectrometers, where SGF suffers abrupt noise transitions due to fixed-window smoothing, WF improved resolution by 40% through SNR-adaptive suppression.^172^ Unlike SGF’s static polynomial fitting, WF dynamically adjusts to local SNR conditions—proving indispensable for applications like motion artifact removal in NIR spectroscopy.

### Kalman filtering

While WF provides an optimal frequency-domain solution for stationary processes, Kalman filtering (KF)^173^ extends this framework to non-stationary signals by recursively updating state estimates in real-time.

The KF algorithm operates via two core stages: the time update (prediction) and measurement update (correction). Time update propagates the prior state estimate ${\hat{x}}_{k-1}$ and error covariance $P_{k-1}$ forward equations^174^^,^^175^:

(Equation 98) $$\left\{ \begin{array}{cc} \text{State}\;\text{prediction:} & x_{k}^{-}=A{\hat{x}}_{k-1}\\ \text{Prior}\;\text{Covariance:} & P_{k}^{-}=AP_{k-1}A^{T}+Q \end{array}\right.$$

Here, $x_{k}^{-}$ and $P_{k}^{-}$ represent the predicted state and error covariance, respectively, with A as the state transition matrix and Q as process noise covariance.

Measurement update integrates new observations $z_{k}$ to refine predictions:

(Equation 99) $$\left\{ \begin{array}{cc} \text{Measurement}\;\text{equation:} & z_{k}=Hx_{k}+v_{k}\\ \text{Residual:} & y_{k}=z_{k}-H{\hat{x}}_{k}^{-}\\ \text{Kalman}\;\text{gain:} & K_{k}=P_{k}^{-}H^{T}{\left(HP_{k}^{-}H^{T}+R\right)}^{-1}\\ \text{Posterior}\;\text{state:} & {\hat{x}}_{k}={\hat{x}}_{k}^{-}+K_{k}y_{k}\\ \text{Posterior}\;\text{covariance:} & P_{k}=\left(I-K_{k}H\right)P_{k}^{-} \end{array}\right.$$

Here, H maps state to measurement space, $v_{k}$ is measurement noise (covariance R), and Kalman gain $K_{k}$ optimally balances prediction and observation. The covariance matrices $P_{k}^{-}$ and $P_{k}$ quantify estimation errors ($e_{k}^{-}$, $e_{k}$):

(Equation 100) $$\left\{ \begin{array}{ccc} P_{k}^{-}=E\left[e_{k}^{-}{\left(e_{k}^{-}\right)}^{T}\right]\text{,} & e_{k}^{-}=x_{k}^{-}-{\hat{x}}_{k}^{-}\text{,} & \text{Prior}\\ P_{k}=E\left[e_{k}{\left(e_{k}\right)}^{T}\right]\text{,} & e_{k}=x_{k}-{\hat{x}}_{k}\text{,} & \text{Posterior} \end{array}\right.$$

KF recursively minimizes the posterior error covariance $P_{k}$ in the minimum mean square error (MMSE) sense, adapting dynamically to new data. KF’s versatility is demonstrated across spectroscopic techniques, where it enhances precision by fusing model predictions with noisy sensor data.

The recursive nature of KF allows it to adapt to dynamic systems, making it particularly effective in spectroscopic applications where real-time noise suppression and drift correction are critical. This is demonstrated in the following cases. In NO_2_ cavity ring-down spectroscopy, it improved detection limits 9.12-fold by suppressing transient noise.^176^ For glucose Raman spectroscopy, it reduced PLS model RMSE from 0.38 to 0.17 g/L. In LIBS steel analysis,^177^ it corrected instrumental drift, lowering Mn prediction RSD from 35% to 11%.^178^ Remarkably, for QCL CO_2_ isotopologues, it achieved δ^13^C (−9.19 ± 0.29‰) and δ^18^O (−1.46 ± 0.34‰) measurements with 7-fold better precision in just 1 s, matching 95-s averaging.^179^

### Neural network adaptive filter

While KF provides optimal state estimation under linear Gaussian assumptions, Neural Network Adaptive Filtering (NNAF)^180^^,^^181^ extends this framework by learning nonlinear dynamics and noise characteristics directly from data, enabling robust performance in complex environments. The NNAF method combines the stability of least mean squares (LMS) filtering with the adaptability of neural networks through backpropagation optimization, where key parameters are carefully designed to balance convergence speed and steady-state error.

The core algorithm operates through three fundamental components:

First (Error Calculation), the instantaneous prediction error quantifies the discrepancy between the desired signal and filter output^182^:

(Equation 101) $$e\left(n\right)=d\left(n\right)-X^{T}\left(n\right)W\left(n\right)$$

where d(n) represents the desired signal (reference measurement), X(n) denotes the input vector (current signal observations), and W(n) is the adaptive weight vector containing the filter coefficients.

Next (Weight Update Mechanism), the filter coefficients are adjusted using a modified LMS rule:

(Equation 102) $$W\left(n+1\right)=W\left(n\right)+ue\left(n\right)X^{T}\left(n\right)$$

The adaptive learning factor u dynamically controls the update magnitude, preventing instability while maintaining fast convergence.

Finally (Adaptive Step Size Control), the critical adaptation component u is governed by:

(Equation 103) $$u=\left\{ \begin{array}{cc} u_{\max}, & \beta >u_{\max}\\ u_{\min}, & \beta <u_{\min}\\ \beta , & \text{otherwise} \end{array}\right.$$

To ensure the convergence, β must satisfy $\beta <n/\lambda_{\max}$, where $\lambda_{\max}$ is the maximum eigenvalue and is positive. To speed up convergence, the core adaptation logic combines a sigmoid-shaped controller (instantaneous error function) and an error-driven exponential term (error factor):

(Equation 104) $$u=\left(-0.5+\frac{1}{1+e^{-a\left|x\right|}}\right)\left(e^{{\left|x\right|}^{r}}-1\right)$$

The term $1/\left(1+e^{-a\left|x\right|}\right)$ regulates adaptation sensitivity, where parameter a controls steepness. The term $e^{{\left|x\right|}^{r}}-1$ introduces nonlinear error-driven adjustments, with exponent r governing growth intensity.

The NNAF demonstrates superior denoising capabilities in practical applications, including spectroscopic analysis: achieved an SNR improvement of 3–4 dB in predicting copper and cobalt impurity concentrations in UV-Vis spectra during wet zinc extraction processes; and prediction accuracy: delivered a goodness-of-fit (R^2^) exceeding 0.99^182^, confirming highly reliable modeling performance.

### Wavelet shrinkage denoising method

While NNAF relies on learned nonlinear mappings for adaptive denoising, wavelet shrinkage denoising (WSD) employs transform-domain processing, offering computationally efficient and theoretically optimal noise removal through thresholding of sparse wavelet representations. WSD is particularly effective for piecewise-smooth signals with transient features, where noise and signal components can be separated via rigorously derived thresholds. A standard approach is the universal threshold from VisuShrink,^183^^,^^184^ defined as:

(Equation 105) $$\lambda =\sigma_{j}^{Noise}\sqrt{2\;\ln\;N}$$

where $\sigma_{j}^{Noise}$ estimates the noise standard deviation at decomposition level j, and N is the signal length. For improved adaptability, level-dependent thresholds may be used^185^:

(Equation 106) $$\lambda_{j}=\sigma_{j}^{Noise}\sqrt{2\;\log_{2}\left(N_{j}\right)}$$

where $N_{j}$ represents the number of coefficients at level j. More sophisticated methods apply locally adaptive thresholds based on coefficient statistics^184^:

(Equation 107) $$\begin{array}{cc} \lambda_{j,L}=\mu_{j}-\kappa_{j,L}\sigma_{j}\text{,} & \lambda_{j,H}=\mu_{j}+\kappa_{j,H}\sigma_{j} \end{array}$$

where, $\mu_{j}$ and $\sigma_{j}$ denote the mean and standard deviation of wavelet coefficients at level j:

(Equation 108) $$\begin{array}{cc} \mu_{j}=\sum_{i=1}^{N_{j}}w_{j,i}/N_{j}\text{,} & \sigma_{j}=\sqrt{\frac{1}{N_{j}-1}\sum_{i=1}^{N_{j}}{\left(w_{j,i}-\mu_{j}\right)}^{2}} \end{array}$$

Thresholds are applied via hard thresholding:

(Equation 109) $${\tilde{w}}_{j,i}=\left\{ \begin{array}{ll} w_{j,i} & \left|w_{j,i}\right|\geq \lambda_{j}\\ 0 & \left|w_{j,i}\right|<\lambda_{j} \end{array}\right.$$

or soft thresholding, which ensures smoother transitions:

(Equation 110) $${\tilde{w}}_{j,i}=\text{sgn}\left(w_{j,i}\right)\cdot \max\left(\left|w_{j,i}\right|-\lambda_{j},0\right)$$

where sgn(·) preserves the coefficient’s sign.

The denoising process involves: Selecting a wavelet basis (e.g., Daubechies and Symlet), Decomposing the signal to an optimal level $k=\left[\log_{2}\;N\right]$, Estimating $\sigma_{j}^{Noise}$ (e.g., via MAD), thresholding detail coefficients, and reconstructing via inverse discrete wavelet transform (IDWT).

This approach demonstrates remarkable performance: In THz-TDS measurements of GFRPs, it reduced thickness errors from 16.4% to 3.7%.^186^ For ESR spectroscopy, it achieved 32 dB SNR improvement versus conventional methods (10 dB).^184^

The method’s strength lies in its statistically informed thresholding, adaptively distinguishing signal from noise—crucial for precision-demanding applications like materials science and spectral analysis.

### Hilbert vibration decomposition (HVD)

While WSD employs fixed wavelet bases for noise suppression, Hilbert vibration decomposition (HVD) adaptively extracts signal components through envelope demodulation—leveraging the analytic signal derived from the Hilbert transform of the original signal x(t)^187^^,^^188^^,^^189^:

(Equation 111) $$H\left[x\left(t\right)\right]=\frac{1}{\pi }P.V.\;\int_{-\infty }^{\infty }\frac{x\left(\tau \right)}{t-\tau }d\tau$$

where P.V. denotes the Cauchy principal value. The analytic signal is constructed as:

(Equation 112) $$z\left(t\right)=x\left(t\right)+iH\left[x\left(t\right)\right]=A\left(t\right)e^{i\phi \left(t\right)}$$

with instantaneous frequency ω(t)=dϕ(t)/dt, where A(t) and ϕ(t) represent the instantaneous amplitude and phase respectively. The decomposition algorithm proceeds as follows:

First, extract the dominant component $x_{1}\left(t\right)$ through demodulation with reference frequency $\omega_{r}$:

(Equation 113) $$\left\{ \begin{array}{l} \left\langle x_{1}\left(t\right)\right\rangle =\frac{A_{1}\left(t\right)}{2}\cos\left[\phi_{1}\left(t\right)\right]\\ \left\langle {\tilde{x}}_{1}\left(t\right)\right\rangle =\frac{A_{1}\left(t\right)}{2}\sin\left[\phi_{1}\left(t\right)\right] \end{array}\right.$$

Next, reconstruct the vibrational mode:

(Equation 114) $$x_{1}\left(t\right)=A_{1}\left(t\right)\cos\left[\phi_{1}\left(t\right)\right]$$

Finally, obtain residual signal:

(Equation 115) $$r\left(t\right)=x\left(t\right)-x_{1}\left(t\right)$$

The process repeats on r(t) until standard deviation of successive frequency estimates falls below δ=0.001.^190^

This method has demonstrated remarkable performance in applications such as structural vibration analysis and spectroscopic signal processing, where it can enhance signal-to-noise ratios from 3.53 to 130.64^189^ and improve classification accuracy from 40% to 90.25%.^191^ While particularly effective for oscillatory AM/FM signals, complementary approaches may be required for non-harmonic components.

### Spectroscopic filtering: toward a domain-adaptive framework

Filtering and smoothing methods offer distinct advantages for spectral data preprocessing: GF employs weighted averaging to suppress noise, excelling at high-frequency noise reduction though requiring careful parameterization to avoid over-smoothing; KF, through state-space modeling, provides optimal recursive estimation for dynamic systems with time-varying signals, while WF operates in the frequency domain to minimize mean-square error by leveraging signal-to-noise statistics in stationary processes. For complex datasets, the neural network adaptive filter (NNAF) combines LMS stability with neural network flexibility for robust denoising, whereas Savitzky-Golay filtering preserves peak integrity via local polynomial regression to balance noise removal and feature retention. Meanwhile, WSD exploits signal sparsity in multiscale domains through adaptive thresholding, and HVD specializes in nonstationary multicomponent signal analysis via instantaneous frequency extraction. Ultimately, the choice among these techniques hinges on noise characteristics, signal dynamics, and the critical trade-off between smoothing intensity and feature fidelity.

## Spectral derivatives

While smoothing suppresses noise in spectral data, it often obscures subtle features. Spectral derivative preprocessing^22^ addresses this by computing derivatives to enhance slope variations while suppressing baseline drift. Unlike smoothing, derivatives sharpen inflection points, improving spectral resolution—critical for hyperspectral imaging and vibrational spectroscopy. This approach balances noise resilience with feature discrimination. Derivative techniques amplify weak spectral features while reducing noise and scattering artifacts. Their effectiveness depends on the differentiation method, which governs the resolution-noise tradeoff. We systematically evaluate four key derivative methods by their mathematical sophistication and practical utility.

### Finite difference method

As the most fundamental numerical differentiation approach, the finite difference method (FDM) computes derivatives by approximating discrete differences between adjacent spectral points. Assuming the spectral signal is sampled at discrete points $x_{i}$, with a constant sampling interval h, FDM can be mathematically expressed as follows^22^:

(Equation 116) $$\left\{ \begin{array}{l} \begin{array}{cc} y_{i}^{\prime }=\frac{y_{i}-y_{i-1}}{h}, & h=x_{i+1}-x_{i} \end{array}\\ y_{i}^{\prime \prime }=\frac{y_{i+1}-2y_{i}+y_{i-1}}{h^{2}} \end{array}\right.$$

FDM approximates the first derivative $y_{i}^{\prime }$ by calculating the difference between consecutive spectral data points $y_{i}$ and $y_{i-1}$, normalized by the sampling interval h. Similarly, the second derivative $y^{\prime \prime }$ is computed using a centered difference formula involving $y_{i+1}$, $y_{i}$, and $y_{i-1}$. While computationally efficient, FDM suffers from inherent noise sensitivity, where minor fluctuations lead to amplified derivative artifacts. Truncation errors further limit accuracy, scaling linearly (O(h)) and quadratically ($O\left(h^{2}\right)$) for the first and second derivatives, respectively. This makes FDM primarily useful for clean, high-resolution spectra where computational simplicity is prioritized over robustness.

### Savitzky-Golay derivatives

An extension of FDM, the Savitzky-Golay derivatives (SGD) addresses noise amplification by incorporating polynomial smoothing prior to differentiation.^192^^,^^193^ The SGD method operates by selecting a window of n=2m+1 (where n typically ranges up to 25) consecutive points within a spectral interval of N points, with the central point designated as $u_{i}$. These points are initially fitted to a p-th order polynomial, $Y_{j}$, expressed as follows:

(Equation 117) $$Y_{j}=\sum_{q=0}^{p}c_{q}j^{q}=a_{0}+a_{1}j+\ldots +a_{p}j^{p}$$

Then, the coefficients corresponding to its various derivatives are derived through polynomial fitting. Subsequently, a least squares fitting procedure is applied to the spectrum $Y_{j}$ within the window, with the fitting error denoted as S:

(Equation 118) $$S=\sum_{j=-m}^{m}{\left(Y_{j}-y_{j}\right)}^{2}$$

Minimization of this error is achieved by solving the partial derivative condition $\partial S/\partial a_{k}=0$. Curve smoothing in SGD is centered at j=0, where a set of coefficients $\left\{p_{s}^{\left(0\right)}\right\}$ is determined to compute the smoothed value ${\bar{u}}_{i}$ of the i-th point. Alternatively, distinct sets of coefficients $\left\{p_{s}^{\left(q\right)}\right\}$ can be utilized to calculate the smoothed values of the q-th derivative, as represented by $d^{q}{\bar{u}}_{i}/dx^{q}$, where i=m+1,…,N−m−1:

(Equation 119) $$\begin{array}{cc} {\bar{u}}_{i}=\sum_{s=-m}^{m}p_{s}^{\left(0\right)}u_{i+s}\text{,} & d^{q}{\bar{u}}_{i}/dx^{q}=\sum_{s=-m}^{m}p_{s}^{\left(q\right)}u_{i+s} \end{array}$$

(Equation 120) $$p_{s}^{\left(0\right)}=\frac{3\left[3m^{2}+3m-1-5s^{2}\right]}{\left(2m+3\right)\left(2m+1\right)\left(2m-1\right)}$$

(Equation 121) $$p_{s}^{\left(1\right)}=\frac{3s}{\left(2m+1\right)\left(m+1\right)\left(m\right)}$$

(Equation 122) $$p_{s}^{\left(2\right)}=\frac{30\left[3s^{2}-m\left(m+1\right)\right]}{\left(2m+3\right)\left(2m+1\right)\left(2m-1\right)\left(m+1\right)\left(m\right)}$$

where, $p_{s}^{\left(0\right)}$ and $p_{s}^{\left(2\right)}$ correspond to the coefficients for the zero and second derivatives of SGD, respectively, obtained through fitting with second- or third-order polynomials. Similarly, $p_{s}^{\left(1\right)}$ denotes the coefficient for the first derivative of SGD, derived from a second-order polynomial fit.^194^

The Savitzky-Golay (SG) filter shares a convolution-based approach with mean filtering but includes polynomial fitting for simultaneous smoothing and derivative computation. Its ability to preserve spectral features makes it particularly effective in classification tasks; for instance, SG first derivatives paired with SVM classifiers achieved 100% accuracy in NIR-based wood species identification.^195^ In baseline correction of Rhodiola FT-NIR spectra, the Norris-Williams (NW) derivatives showed slightly higher $R^{2}$ values (98.5 vs. 96.5 for first derivatives; 97.4 vs. 93.1 for second derivatives), but SG derivatives offer greater flexibility in optimizing window size and polynomial order.^196^ SG second derivatives excel in crystallization monitoring, balancing noise suppression, feature enhancement, and computational efficiency for real-time analysis.^197^ The method’s strength lies in its dual smoothing-differentiation capability, which retains spectral features better than simple filters. However, performance hinges on careful parameter selection (polynomial order, window size), requiring dataset-specific tuning.

### Norris-Williams derivatives

Like SGD, the Norris-Williams derivatives (NWD)^198^^,^^199^ integrates smoothing with derivative computation but replaces polynomial regression with a moving-average filter. Assuming spectral signals are sampled at discrete points $x_{i}$ with a constant interval h, the method constructs its mathematical model as follows:

(Equation 123) $$\left\{ \begin{array}{l} {\bar{y}}_{i}=\frac{1}{2m+1}\sum_{j=-m}^{m}y_{i+j}\\ \begin{array}{cc} y_{i}^{\prime }=\frac{{\bar{y}}_{i}-{\bar{y}}_{i-1}}{h}, & y_{i}^{\prime \prime }=\frac{{\bar{y}}_{i+1}-2{\bar{y}}_{i}+{\bar{y}}_{i-1}}{h^{2}} \end{array} \end{array}\right.$$

In this method, spectral smoothing is first achieved by averaging 2m+1 points within a window centered on measurement point i, where m defines the window size. The smoothed signal ${\bar{y}}_{i}$ then serves as the basis for derivative calculations. The first derivative $y_{i}^{\prime }$ is computed as the difference between consecutive smoothed values (spaced by h), while the second derivative $y_{i}^{\prime \prime }$ incorporates a second-order difference to capture curvature.

The NWD method effectively combines smoothing with gap-size adjustment to reduce noise and improve the signal-to-noise ratio (SNR), outperforming traditional finite difference methods (FDMs). It excels in detecting segregation in particulate systems, particularly for trace components (e.g., enzyme placebo granules in complex mixtures). Unlike standard first/second derivatives, SNV, or SG smoothing, NWD minimizes noise interference while preserving spectral details, making it particularly effective for low-concentration detection.

In NIR spectral analysis, NWD achieved a high segregation index (0.71) with minimal error (low MAE and MAPE), proving reliable for industrial quality control.^199^ However, NWD’s performance depends on optimizing the smoothing window (m) and gap size (h) for each application. Its simplicity enables fast computation—beneficial for real-time industrial processing.

Compared to SG’s polynomial flexibility, NWD’s uniform smoothing may occasionally obscure fine spectral features. Nonetheless, it remains superior in applications prioritizing noise suppression and rapid analysis, as demonstrated in enzyme granule segregation studies.

### Grunwald-Letnikov fractional order derivation

Building on NWD’s robustness of in industrial applications, the Grunwald-Letnikov fractional-order derivative (GL-FOD) enhances spectral processing flexibility through fractional-order differentiation. By tuning the derivative order α, GL-FOD optimizes feature resolution for complex spectral datasets—capturing nonlinear patterns inaccessible to integer-order derivatives. For a continuous function (x) , GL-FOD is rigorously defined as^200^^,^^201^^,^^202^:

(Equation 124) $$d^{\alpha }f\left(x\right)=\lim_{h\to 0}\frac{1}{h^{\alpha }}\sum_{m=0}^{\left[\frac{b-a}{h}\right]}\frac{{\left(-1\right)}^{m}\Gamma \left(\alpha +1\right)}{\Gamma \left(m+1\right)\Gamma \left(\alpha -m+1\right)}f\left(x-mh\right)$$

where h is the step size, a and b are the lower and upper limits of differentiation, [(b−a)/h] refers to the integer part of (b−a)/h, and Γ(z) denotes the gamma function for interpolation, given by:

(Equation 125) $$\Gamma \left(z\right)=\int_{0}^{+\infty }u^{z-1}e^{-u}du=\left(z-1\right)!$$

For discrete spectral signals $y=\left[y_{1},y_{2},\cdots ,y_{n}\right]$, the discrete implementation uses an n×n fractional differential matrix $D^{\alpha }$:

(Equation 126) $$\begin{array}{cc} D^{\alpha }=\frac{1}{h^{\alpha }}\left[\begin{array}{ccccccc} d_{1} & 0 & \cdots & 0 & \cdots & 0 & 0\\ d_{2} & d_{1} & 0 & \ddots & \ddots & \ddots & \vdots \\ \vdots & \ddots & \ddots & \ddots & 0 & \ddots & 0\\ d_{k} & \cdots & d_{2} & d_{1} & 0 & \ddots & \vdots \\ 0 & \ddots & \ddots & \ddots & \ddots & \ddots & 0\\ \vdots & \ddots & d_{k} & \cdots & d_{2} & d_{1} & 0\\ 0 & \cdots & 0 & d_{k} & \cdots & d_{2} & d_{1} \end{array}\right]\text{,} & d_{k}=\frac{\Gamma \left(k-\alpha \right)}{\Gamma \left(k+1\right)\Gamma \left(-\alpha \right)} \end{array}$$

where the series truncation at k=20 (due to $d_{k}\approx 0$ for k≫1) balances accuracy and computational efficiency.

GL-FOD has demonstrated remarkable efficacy in extracting nonlinear features from hyperspectral data, significantly enhancing analytical accuracy across diverse applications. In agriculture, GL-FOD coupled with PLS regression achieved an $R^{2}$ of 0.84 for cadmium detection in rice leaves,^201^ while a 0.6-order derivative combined with random forest regression improved soil organic matter prediction ($R^{2}=0.66$).^62^ For winter wheat monitoring, a 0.3-order derivative with CARS-ETsR modeling attained a validation $R_{v}^{2}$ of 0.8667 in chlorophyll density estimation, outperforming integer-order derivatives by 17% in Na^+^ correlation enhancement.^203^ Notably, in reservoir characterization, GL-FOD’s recursive formulation with Hausdorff accumulation kernels demonstrated superior volatility modeling and long-term trend capture (higher $R^{2}$, lower RMSE versus conventional models).^204^

### Spectral derivatives: balancing resolution and noise

The four derivative approaches—FDM, NWD, SG, and GL-FOD—offer distinct advantages and trade-offs in spectral analysis. FDM provides straightforward implementation but suffers from significant noise sensitivity, limiting its utility in low-SNR environments. NWD (Norris-Williams derivatives) excels in noise suppression and real-time processing, making it particularly suitable for industrial quality control where baseline stability is critical. SG (Savitzky-Golay) achieves a balance between smoothing and feature preservation through polynomial fitting, though its fixed-window design restricts adaptability to dynamic spectral features. In contrast, GL-FOD leverages fractional calculus to enable fine-tunable differentiation, delivering superior multi-scale resolution for complex datasets such as nonlinear mineral-organic mixtures. While NWD and SG are efficient for routine applications, GL-FOD represents a more advanced solution when precision and flexibility are paramount. Ultimately, method selection depends on the interplay of noise tolerance, computational efficiency, and spectral detail requirements—with GL-FOD emerging as the preferred choice for applications demanding high feature discrimination.

## Data information mining

Conventional spectral preprocessing relies on removal-based techniques (e.g., noise filtering, baseline correction), often overlooking intrinsic data relationships. In contrast, correlation-driven approaches (e.g., 2D/3D correlation) exploit interdependencies among spectral points to extract latent information.

The three-dimensional correlation method (3dCM)^205^ enhances spectral analysis through sequential Hilbert transforms (HT) and tensor product. Given a spectral dataset with q sample types, each type k contains $s_{k}$ spectra (total samples $s=\sum_{k=1}^{q}s_{k}$) and w frequency points (matrix $M\in R^{s\times w}$). The process involves three key steps:

Step 1: Orthogonal Signal Generation via HT. For each sample type k, apply HT to spectral matrix $M^{\left(k\right)}$ to obtain a 90° phase-shifted matrix $H^{\left(k\right)}$:

(Equation 127) $$H^{\left(k\right)}=H\left[M^{\left(k\right)}\right]=\frac{1}{\pi }\int_{-\infty }^{\infty }\frac{M^{\left(k\right)}\left(\tau \right)}{t-\tau }d\tau$$

Step 2: 2D Correlation Matrix Construction. The tensor product of $M^{\left(k\right)}$ and $H^{\left(k\right)}$ generates a 2D correlation matrix $X^{\left(k\right)}$ (2dCM^206^):

(Equation 128) $$X_{ij}^{\left(k\right)}=\sum_{m=1}^{s_{k}}H_{im}^{\left(k\right)}{\left[M_{mj}^{\left(k\right)}\right]}^{T}$$

After vectorization to $Y^{\left(k\right)}$ ($Y\in R^{s^{\prime }\times w^{\prime }}$), a second HT is applied to produce ${H^{\prime }}^{\left(k\right)}$:

(Equation 129) $${H^{\prime }}^{\left(k\right)}=H\left[Y^{\left(k\right)}\right]$$

Step 3: 3D Tensor Expansion. The final 3D correlation matrix $Z^{\left(k\right)}$ is computed by:

(Equation 130) $$Z_{ij}^{\left(k\right)}=\sum_{m^{\prime }=1}^{s^{\prime }}\sum_{m=1}^{s}{H^{\prime }}_{im^{\prime }}^{\left(k\right)}{\left[M_{mj}^{\left(k\right)}\right]}^{T}$$

Vectorization of $Z_{ij}^{\left(k\right)}$ yields $W^{\left(k\right)}$ ($W\in R^{s^{\prime \prime }\times w^{\prime \prime }}$)with cubically expanded resolution ($w^{\prime \prime }=w^{3}$) and augmented sample size $s^{\left(3\right)}$:

(Equation 131) $$\begin{array}{cc} s^{\left(3\right)}=\sum_{k=1}^{q}\left(\sum_{i=1}^{s_{k}^{\left(2\right)}-1}\sum_{j=1}^{s_{k}}j\right)\text{,} & s_{k}^{\left(2\right)}=\sum_{i=1}^{s_{k}-1}\sum_{j=i+1}^{s_{k}}j \end{array}$$

In practical applications like Chinese handmade paper authentication, this preprocessing boosted machine learning accuracy from <10% to >99% across PCA-LR, PLS-LR, KNN, RF, and CNN models by effectively increasing both spectral resolution and training data volume.^142^

## Discussion and conclusion

The evolution of spectral preprocessing has progressed from basic noise removal to sophisticated information enhancement strategies, with modern techniques now targeting specific analytical challenges through optimized approaches. Cosmic ray removal exemplifies the precision-speed trade-off, spanning rapid moving average filtering to advanced WSD and kernel PCA residual diagnosis (KPCARD), while baseline correction has advanced from polynomial fitting to adaptive asymmetric least squares (AsLS) and interpretation-guided DL models. Scattering correction techniques like MSC and its extended variant (EMSC)—especially effective for biological samples via combined PCA denoising—along with SNV normalization demonstrate method specialization for distinct interference types. Normalization methods such as *Z* score standardization and L2 normalization provide fundamental variance stabilization, whereas derivative analysis now benefits from fractional-order differentiation (GL-FOD)’s superior multicomponent resolution compared to traditional finite differences.

Conventional spectral preprocessing relies on subtraction-based techniques (e.g., noise reduction and baseline correction), which often compromise underlying data correlations. In contrast, emerging correlation-based methods—particularly three-dimensional correlation analysis (3dCM)—explicitly preserve spectral interdependencies while providing deeper insights into spectral relationships. By leveraging Hilbert transforms and tensor operations, this approach expands spectral resolution from [w] to [$w^{3}$] dimensions, significantly enhancing feature discrimination, as evidenced by benchmark classification accuracy improvements from <10% to >99% in material authentication studies.

Challenges remain, however: manual parameter tuning impedes high-throughput implementation, computational demands vary widely (from efficient MAF to intensive WSD/KPCARD), and the opacity of DL models hinders interpretability. Addressing these limitations is critical for broader adoption of advanced spectral analysis.

Future progress in spectroscopic data preprocessing will depend on three critical advancements.

(1) Context-aware algorithms capable of dynamic adaptation to varying data conditions;
(2) Hybrid modeling frameworks that integrate physics-based constraints (e.g., correlation-preserving methods such as 3dCM) to enhance the robustness of data-driven approaches;
(3) Reinforcement learning-driven automation to optimize the trade-off between computational efficiency and information fidelity.

These innovations—illustrated, for example, by 3dCM’s Hilbert-transform-enhanced tensor operations (improving classification accuracy from <10% to >99% in benchmark cases)—signal a paradigm shift from mere distortion correction to structured feature enhancement. By unifying computational efficiency, physical fidelity (via spectral relationship encoding), and autonomous optimization, next-generation preprocessing methods will transform spectroscopic analysis into a powerful, information-enriching platform for both scientific and industrial applications.

### Limitations of the study

This review primarily focuses on high-impact publications to ensure methodological rigor, though we acknowledge that certain niche or industry-specific approaches may receive less coverage, and some methods may be inherently tailored to specific spectral techniques (e.g., Raman vs. NIR) or application domains (e.g., biomedical vs. agricultural). Given the rapid advancements in AI-driven spectral analysis, some cutting-edge developments might not yet be fully reflected in the existing literature. Direct comparisons between preprocessing techniques (e.g., baseline correction vs. smoothing filters) are inherently challenging due to methodological incompatibilities, where each approach is typically optimized and validated against distinct metrics or task-specific objectives. Lab-scale results may require further validation under real-world conditions. While our systematic approach aims to mitigate bias, the broader literature landscape exhibits a natural inclination toward widely cited methodologies, whereas hardware implementation challenges (e.g., computational efficiency and cost) merit deeper exploration in future work. Additionally, the scarcity of embedded-system validations highlights opportunities for extending this research direction in subsequent studies.

## Acknowledgments

This research received no specific grant from funding agencies in the public, commercial, or not-for-profit sectors.

## Author contributions

C.S.Y.: Conceptualization, methodology, investigation, formal analysis, writing – original draft, writing – review and editing.

## Declaration of interests

There are no competing interests.
